# Factors associated with 1-year health-related quality of life after neck-specific exercise in chronic whiplash-associated disorders: an exploratory secondary analysis of a randomized controlled trial

**DOI:** 10.1186/s12891-026-10581-x

**Published:** 2026-10-13

**Authors:** Anneli Peolsson, Stefan Ljunggren, Gunnel Peterson

**Affiliations:** 1 Department of Health Medicine and Caring Sciences, Unit of Physiotherapy, Linköping University, Linköping, Sweden; 2 Occupational and Environmental Medicine Centre, Department of Health Medicine and Caring Sciences, Unit of Clinical Medicine, Linköping University, Linköping, Sweden; 3 Centre for Clinical Research Sörmland, Uppsala University, Eskilstuna, Sweden

**Keywords:** Whiplash, Spine, Exercise, Rehabilitation, Association, Quality of life

## Abstract

**Background:**

Factors associated with changes in health-related quality of life (HRQoL) outcomes in neck disorders have not been investigated after internet-based support for exercise. The purpose of the study was to investigate baseline and short-term outcome factors associated with changes in HRQoL (EQ-5D-3L and EQ-VAS) at 3 and 15 months, respectively, after a neck-specific exercise programme (NSE) for individuals with chronic whiplash-associated disorders grades 2 and 3.

**Methods:**

This was a secondary analysis of a prospective randomized controlled multi-centre trial with concealed allocation. Participants (n = 140) were randomized to NSE at a physiotherapy clinic or internet-based NSE with four physiotherapy visits (NSEIT). Assessor-blinded investigators obtained measurements at baseline and at 3- and 15-month follow-up. Orthogonal partial least square (OPLS) and OPLS discriminant statistical analyses (OPLS-DA) were used.

**Results:**

There were no significant differences between the exercise programmes. Improvement in psychosocial measures, pain, disability and several objective measures were associated with improvements in HRQoL in OPLS models (R^2^ = 0.20 to 0.27, Q2 = 0.06 to 0.12, p < 0.05 to < 0.001). Higher baseline score on adverse psychosocial measures and disability, together with lower dizziness, self-efficacy, and neck-muscle endurance in extension, were associated with clinically relevant improvement in EQ-5D-3L at 15-month follow-up (R^2^ = 0.26, Q^2^ = 0.13, p < 0.01). The other models were non-significant.

**Conclusions:**

Several factors, including adverse psychosocial measures, may be clinically relevant for HRQoL after neck-specific exercise interventions. Given the models’ weak predictive ability, the findings should be regarded as exploratory and hypothesis-generating.

**Trial registration:**

The study was registered before data collection started, ClinicalTrials.gov Protocol ID: NCT03022812, initial release 12/20/2016.

The study was approved by the regional ethical review board in Linköping, Sweden (Dnr 2016/135–31; 2016/526–32; 2017/45–32).

**Supplementary information:**

The online version contains supplementary material available at https://doi.org/10.1186/s12891-026-10581-x.

## Introduction

Neck pain is among the most disabling conditions in terms of years lived with disability, according to the Global Burden of Diseases, Injuries, and Risk Factors [1]. Individuals with neck pain after a traumatic accident (whiplash-associated disorders, WAD) have reported worse outcomes than those with non-traumatic neck disorders [2]. Those with WAD grade 1 (neck pain, not clinically verified) and grade 2 (musculoskeletal findings during a physical examination verifying patient’s history of pain emanating from the neck) were shown to have a worse outcome compared with those experiencing other mild traumatic traffic accidents [3]. WAD has also been reported to easily transit to chronicity [3–7].

WAD considerably reduces health-related quality of life (HRQoL) due to pain and physical, psychological, and social disability, reduced work ability, and lower income [4, 6, 8–10].

There is a substantial number of studies regarding factors associated to pain and disability in WAD [4, 5, 7], but rather scarce information regarding factors relating to HRQoL. Only Landén Ludvigsson et al. [11] have investigated factors of importance for HRQoL outcomes after a rehabilitation programme. Neck-related factors, both psychosocial and work ability, were associated with change in HRQoL at the 6-month follow-up after a neck-specific exercise programme [11]. It is of utmost importance to improve rehabilitation for those already in the chronic stage, and especially for those with more severe WAD (grade 3, as grade 2 but with additional neurological findings) [12], who are often excluded from intervention studies. To do so, more knowledge about factors associated with the rehabilitation outcome is needed.

In a randomised controlled trial [10], we investigated whether neck-specific exercise with internet support (NSEIT), supplemented by four physiotherapy sessions over a 12-week period, was non-inferior to the same exercise programme delivered under physiotherapist supervision twice weekly for 12 weeks (NSE; a maximum of 24 sessions) in individuals with chronic WAD grades 2 or 3. The results in the study [10] demonstrated significant improvements in HRQoL, with no significant differences between NSE and NSEIT, thereby supporting the non-inferiority of NSEIT. Factors associated with a change in HRQoL outcome in neck disorders have not been investigated after internet-based support for exercise.

The aim of this study was to investigate factors associated to changes in HRQoL after NSEIT or NSE for individuals with chronic WAD grades 2 and 3. Because of the potential intercorrelations among factors, multivariate methods were used to assess their relationship with HRQoL outcomes.

## Methods

The details of the criteria, recruitment process, and neck-specific exercise programmes have been reported elsewhere [10] and are briefly described in the text below.

**Fig. 1:**
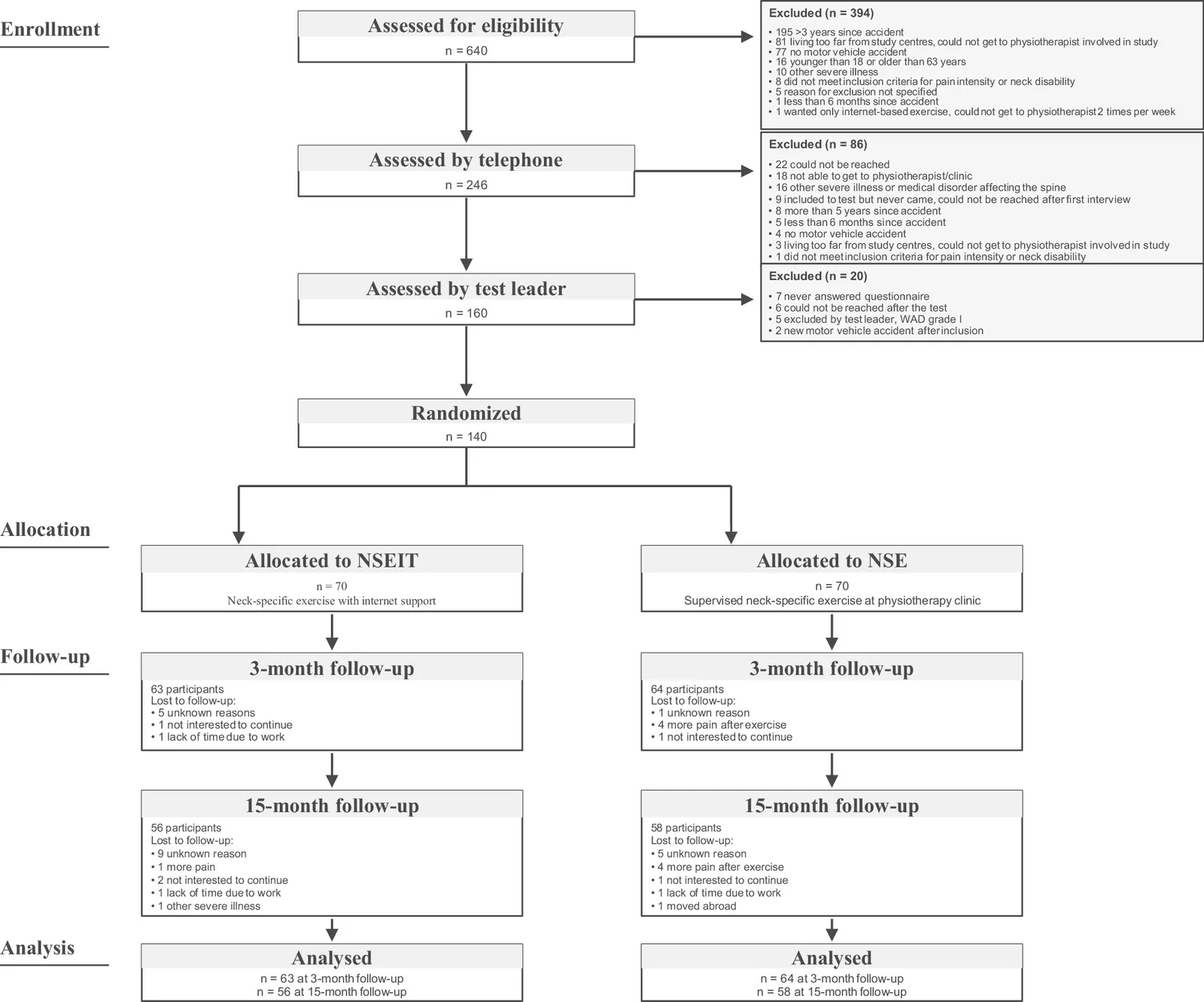
CONSORT flow-diagram of participant enrolment, allocation to study groups, follow-up, and analysis

This is a secondary analysis of an RCT multi-centre non-inferiority trial [10] comparing two different ways of distributing a 3-month neck-specific exercise programme: NSEIT or NSE with additional information. The study follows the Declaration of Helsinki and was approved by the regional ethical review board in Linköping, Sweden (Dnr 2016/135–31; 2016/526–32; 2017/45–32) (please see supplementary (S) Table S1 for the Consort check list, Fig. 1 for Consort flow-chart and Table S2 for the Spirit check list). The study was registered before data collection started (ClinicalTrials.gov Protocol ID: NCT03022812, initial release 12/20/2016, Project investigators: Prof. Anneli Peolsson, co-applicant Assoc. Prof. Gunnel Peterson) and the study protocol was published prior to data collection [13]. Data were collected in outpatient care in Sweden between 6 April 2017 and 15 September 2020. The main outcome data for pain and disability has been published [10]. There were no serious adverse harms or side-effects in the study [10].

### Participants and randomization

Potential participants received information about the study mainly through social media. Individuals interested in participating passed a process of several steps before being included in the present study: 1) Completed a web-based survey with a few questions. 2) An apparently eligible participant was contacted by an experienced physiotherapist for a telephone interview to further verify their medical history, along with their ability and motivation to participate in the exercise programme. 3) A physical examination was conducted by a test-leader (not involved in the treatment) at outpatient care to ensure that the criteria for study participation were met. 4) Informed oral and written consent was obtained from all participants. 5) Participants (Table 1) filled out a digital baseline questionnaire, and physical measurements were performed by the test leader.

**Table 1:** Baseline characteristics of participants with whiplash-associated disorders (WAD) grades 2 and 3, by treatment group

Background variables	NSEIT (n = 70)	NSE (n = 70)
**Age**, mean (SD)	40.4 (11.6)	40.5 (11.4)
**Months since injury**, mean (SD)	27.4 (21.0)	25.2 (15.5)
**Women**, n (%)	55 (79%)	55 (79%)
**Smoking**, yes (%)	6 (9%)	7 (10%)
**Marital status, married or cohabiting**, n (%)	52 (74%)	53 (76%)
**Highest education**, n (%)		
Elementary	1 (1%)	0 (0%)
High school	30 (43%)	39 (56%)
University	35 (50%)	27 (39%)
Other	4 (6%)	3 (4%)
**Worried about personal finances**		
Yes, often n (%)	6 (9%)	2 (3%)
Yes, quite often, n (%)	25 (36%)	21 (30%)
No, seldom, n (%)	39 (56%)	47 (67%)
**Post-traumatic symptoms (PCL-S)**, yes, n (%)	41 (59%)	37 (53%)
Car accident	32 (46%)	20 (29%)
Other or unknown	10 (14%)	17 (24%)
(PCL-S, 17–85), mean (SD)	43.1 (14.4)	40.3 (13.3)
**WAD grade**, n (%)		
Grade 2	46 (66%)	43 (61%)
Grade 3	24 (34%)	27 (39%)
**Expectations of the intervention**, (0–10) median (IQR)		
Expectation NSEIT	8.0 (7.0–9.0)	7.5 (6.0–9.0)
Expectation NSE	8.0 (6.0–10.0)	7.5 (5.75–8.25)
**Adherence to excercises at 3 month**		
≥50% self-reported attendance to exercises, yes/n (%)	46/61 (75%)	59/63, (94%)
**Dependent variables**	**NSEIT (n** = **70)**	**NSE (n** = **70)**
**HRQoL**, EQ-5D-3L, mean (SD)	0.57 (0.29)	0.64 (0.21)
**HRQoL**, EQ-VAS (0–100), mean (SD)	57.7 (18.7)	58.6 (17.0)
**Independet variables**	**NSEIT (n** = **70)**	**NSE (n** = **70)**
**Neck pain intensity now** (VAS 0–100), mean (SD)	34.6 (21.4)	39.7 (22.4)
**Neck pain average** (VAS, 0–100), mean (SD)	44.9 (20.6)	47.6 (21.7)
**Neck pain worst** (VAS 0–100), mean (SD)	59.7 (22.1)	65.3 (19.9)
**Dizziness**, yes, n (%)	42(60%)	40 (57%)
**Dizziness at rest** (VAS 0–100), mean (SD)	17.9 (17.1)	17.3 (20.4)
**Dizziness during activity** VAS (0–100), mean (SD)	37.0 (20.9)	33.9 (25.3)
**Unsteadiness** VAS (0–100), mean (SD)	31.1 (22.4)	30.3 (21.3)
**Current headache intensity** VAS (0–100), mean (SD)	15.9 (18.9)	26.0 (25.6)
**Neck Disability Index (NDI)** (0–100%), mean (SD)	39.4 (12.2)	36.6 (10.8)
**Patient-Specific Functional Scale (PSFS)** (0–10), mean (SD)		
PSFS work	3.9 (2.5)	3.9 (2.2)
PSFS leisure time	3.7 (2.1)	3.6 (2.3)
PSFS physical activity	3.2 (2.5)	3.5 (2.8)
**Symptom satisfaction** (0–7), median (IQR)	4.0 (4.0–6.0)	5.0 (4.0–6.25)
**Exercise level** (1–4), median (IQR)	3.0 (2.0–3.0)	3.0 (2.0–4.0)
**Fear-Avoidance Beliefs Questionnaire (FABQ)** (0–66), mean (SD)		
FABQ-PA	10.2 (5.6)	10.3 (6.8)
FABQ-W	13.5 (9.7)	13.9 (9.9)
**Hospital Anxiety and Depression Scale (HADS)**, mean (SD)		
HADS-Depression (0–21)	6.1 (3.8)	5.4 (3.8)
HADS-Anxiety (0–21)	7.1 (4.4)	6.4 (4.0)
**Pain Catastrophizing Scale (PCS)** (0–52), mean (SD)	18.1 (10.9)	16.6 (11.0)
**Self-Efficacy Scale (SES)** (0–200), mean (SD)	144.1 (41.4)	152.1 (34.3)
**Cognitive Failures Questionnaire (CFQ)** (0–100), mean (SD)	45.7 (18.0)	41.8 (16.9)
**Dynamic hand strength**, kilograms, mean (SD)	30.2 (11.3)	28.6 (9.9)
**Ventral neck muscle endurance**, seconds, mean (SD)	31.3 (28.8)	32.3 (34.7)
**Dorsal neck muscle endurance**, seconds, mean (SD)	138.4 (175.5)	106.0 (143.0)
**Cervical ROM flexion/extension**, degree, mean (SD)	90.7 (27.8)	97.7 (25.5)
**Cervical ROM lateral flexion**, degree, mean (SD)	68.5 (18.2)	65.8 (16.9)
**Cervical ROM rotation**, degree, mean (SD)	104.6 (28.7)	109.6 (23.4)
**Cranio-cervical flexion test (CCFT)**, mm Hg, mean (SD)	23.0 (2.7)	23.1 (4.3)
**Standing one leg, eyes closed (SOLEC)**, seconds, mean (SD)	14.8 (10.2)	15.6 (10.8)

*NSEIT*, Neck-specific exercises with internet support; *NSE*, Neck-specific exercises at physiotherapy clinic; *SD*, Standard deviation; *IQR*, Inter-Quartile Range; *VAS*, Visual Analogue Scale, *WAD*, Whiplash-associated disorders; *EQ-5D-3L*, EuroQol five dimension; *EQ-VAS*, EuroQol vertical VAS

#### Inclusion criteria

Persistent neck pain and disability after a whiplash injury in a 4-wheeled motor vehicle traffic accident at least 6 months, but less than 5 years, previously; whiplash-associated disorders (WAD) corresponding to grade 2 or 3 [12]; age between 18 and 63 years; average estimated neck pain in the last week at least 20 mm on the Visual Analog Scale (VAS) [14, 15]; neck disability > 20% on Neck Disability Index (NDI) [16]; daily access to a computer/tablet/smartphone and the internet; and neck pain and/or neck-related problems within the first week after the injury.

#### Exclusion criteria

Signs of head injury at the time of the whiplash injury, or focal neurological changes in smell and taste; previous fractures or dislocation of the cervical spine; known or suspected serious physical pathology or prior cervical surgery; previous severe neck problems that resulted in sick leave of more than a month in the year prior to the current whiplash injury; generalized pain; other illness or injury that may prevent full participation or are contra-indicated for exercise; lack of knowledge of the Swedish language or participation in the earlier NSE study [11].

#### Randomization and the process of starting NSEIT/NSE

A computer-based block randomization list stratified by sex was used to randomize participants to NSEIT or NSE and allocated by a researcher not otherwise involved in any of the tests or treatments. Group allocation and contact details were sent by a researcher in a sealed, opaque envelope to the treating physiotherapist (located near the participant’s home or workplace), who called the participant to book an appointment and to start NSEIT or NSE.

The test leaders were blinded to randomization, and questionnaires were administered and physical measurements performed at baseline and at the 3- and 15-month follow-ups. The participant was blinded to group allocation when baseline data was collected, but for obvious reasons not at follow-ups (Fig. 1).

### Intervention

The intervention was delivered in outpatient settings close to participants’ homes or workplaces, enhancing external validity. Before the study started, the treating physiotherapists (n = 57 from 10 county councils) in outpatient care received 1 day of theoretical and practical training from the project leaders, together with comprehensive oral and written instructions. The physiotherapists were able to contact the project leaders if they required further advice regarding the interventions.

Both programmes contained the same information, the same exercise program and continued for 12 weeks, but were distributed in two different ways. Participation/ exercise was registered in diaries.

#### NSEIT group

The NSEIT group [10] had 4 sessions at the physiotherapy clinic. The first session (week 1) consisted of a clinical examination and an introduction to the initial exercises. In the follow-up sessions (weeks 2, 3, and 7), new exercises were introduced, progressed, and followed up to ensure correct performance. The exercises were individually progressed within a clear framework of options. Both the exercise programme (photos, videos, and text) and additional information about the neurophysiological processes underlying chronic pain, whiplash injury mechanism, neck muscle function, why to exercise the neck, and how to handle pain-relapse were accessible via an internet-based programme. Pain provocation was not allowed. The physical programme started with exercises to activate the deep neck muscle layers and to increase postural control, followed by progression of neck muscle endurance exercises. The participants were allowed to contact the physiotherapist if pain or other symptoms increased during the period. Participants exercised every day outside healthcare during the initial phase (postural control and deep neck muscle activation) and twice/week in the last phase (when performing endurance exercises) outside healthcare. The physiotherapist reported compliance with the appointments at the physiotherapy clinic and participants filled in an exercise diary [10]. When the 3-month exercise programme in the study was completed, participants were encouraged to continue with the exercises on their own.

#### NSE group

The NSE group [10] received the same exercise programme and the same information as the NSEIT group, but it was delivered at the physiotherapy clinic. Home exercises were included, especially during the initial stage when performing exercises to activate the deep neck muscles. The participants attended 2 sessions a week for 12 weeks at a physiotherapy clinic (a maximum of 24 occasions).

### Included variables

Measurements were obtained by assessor-blinded investigators. Validated Swedish translations of the questionnaires were used.

*Dependent factors* (Y-variables; dependent/response variables): HRQoL consisting of the Euroqol five dimensions (EQ-5D-3L; EQ-5D) (range -0.624–1) and EQ vertical VAS (EQ-VAS) 0–100 mm, with higher values indicative of better HRQoL [17, 18] were measured at baseline and at 3- and 15-month follow-ups.

*Background data*: age (years); sex (male/female); smoking (yes/no); level of education; pain duration (months since injury); post-traumatic stress reactions using the post-traumatic stress disorder (PTSD) checklist, consisting of four subscales (re-experience, avoidance, negative alterations in cognition and mood, and hyper-arousal), with a total score ranging from 0 to 80, with higher scores indicating greater PTSD (PCL-S) [19, 20]; WAD grade according to the Quebec Task Force (QTF) classification [12]; Expectations of the intervention on a 0 to 10 scale, with higher scores indicated stronger belief that the treatment would help; type of intervention: NSEIT or NSE [10]. At 3 months follow-up adherence was registered, defined as at least 50% self-reported attendance to exercises [10].

*Independent factors* (X-variables; independent/explanatory variables) obtained at baseline and at 3- and 15-month follow-ups:

#### Pain variables and dizziness

Current neck pain intensity on a VAS (0–100 mm, where 0 = no pain and 100 = worst imaginable pain) [14, 15]; dizziness at rest and during activity, unsteadiness and current headache intensity measured on VAS scales (0–100 mm) [14, 15].

#### Self-rated disability

NDI, self-perceived disability in everyday life due to neck pain, includes 10 items of neck-related disability, which are summed and transformed into a percentage (0%, no pain or disability; 100%, highest score for pain and disability) [16], and Patient-Specific Functional Scale (PSFS) measuring physical function graded from 0 (unable to do) to 10 (functional level equal to preinjury status) regarding self-perceived physical activity, leisure time, and work [21]. For this study, an average of the physical activity, leisure time, and work were used, termed PSFS-avg.

#### Symptom satisfaction

Symptom satisfaction was measured on a 7-point scale of 1 (happy about symptoms) to 7 (unhappy about symptoms) [22].

#### Exercise level

Exercise level rating daily activities and sports (1 = inactivity, 4 = high activity level) [23].

#### Psychological factors and cognitive failure

Operating fear according to the Fear-Avoidance Beliefs Questionnaire (FABQ) consisting of two subscales (Physical Activity Subscale, PA (4 items) and Work Subscale (7 items)) with total scoring of 0 to 66, where higher scores indicate higher fear-avoidance beliefs [24, 25]; depression and anxiety measured with the Hospital Anxiety and Depression Scale (HAD), scoring 0–21 for either anxiety or depression, with higher scores indicating more psychological illness [26]; extent of catastrophizing according to the Pain Catastrophizing Scale (PCS), consisting of three subscales (rumination, magnification, and helplessness) with a total score range of 0–52 with higher scores indicating higher pain catastrophizing [27], Self-Efficacy Scale (SES), self-perceived performance of 20 everyday functional tasks in spite of pain, with a total score range of 0–200, with higher scores indicating higher perceived self-efficacy, i.e. higher confidence in performance [28, 29]; and Cognitive Failures Questionnaire (CFQ), measuring behavioural problems associated with attentiveness and memory in everyday life, with total scores ranging from 0–100, where higher scores indicate a greater frequency of cognitive failures [30].

#### Measures performed by a physiotherapist

Dynamic hand strength measured in kilograms using the Jamar dynamometer [31]; ventral and dorsal neck muscle endurance (NME) registered in seconds [23]; active range of neck motion (AROM) in sagittal, lateral, and frontal planes recorded in degrees using the plastic helmet Cervical range of motion instrument (CROM) [32]; sensorimotor control of the deep cervical flexor muscles during the cranio-cervical flexion test (CCFT), the Stabilizer registered in mm Hg [33]; and balance test standing on one leg with eyes closed measured in seconds (SOLEC) [34].

### Statistical analyses

Multivariate data analysis, using SIMCA software (v.18.0.0.372, Sartorius Stedim Data Analytics AB, Göttingen, Germany), was employed to investigate the associations between variables.

Orthogonal partial least squares (OPLS) and OPLS discriminant analysis (OPLS-DA) were selected because the analysis included numerous potentially correlated variables. It accommodates multicollinearity and missing data while identifying the variables and patterns most strongly associated with the outcome, thereby offering advantages over conventional regression. These model the association between independent variables (X variables) and dependent/response variables (Y-variables) by summarizing the information into a few latent variables (components). This approach allows the identification of associations between multiple X and Y variables simultaneously and separates variation in the independent variables into variation that is associated with and variation unrelated (orthogonal) to the outcome variables. By analysing multiple correlated variables simultaneously, these methods can handle multicollinearity more effectively than traditional regression approaches while reducing model complexity, thereby facilitating interpretation. Missing data were handled using the default NIPALS-based algorithm implemented in SIMCA, which allows estimation of OPLS and OPLS-DA models in the presence of incomplete observations without prior imputation. No additional imputation procedures were performed. No observations or variables were excluded based on the proportion of missing data. An overview of missing data proportions at different time points can be seen in Table S3.

First, differences between patients randomized to either NSEIT or the same exercises and information at a physiotherapy clinic (NSE) were investigated by studying the changes (change scores) of variables between three time points (baseline to 3 months, baseline to 15 months, and 3 months to 15 months) using OPLS-DA. Both dependent factors (Y-variables; EQ-5D and EQ-VAS) and independent factors were included as X variables to investigate which variables may explain differences between NSE and NSEIT.

Second, OPLS models were used to investigate the association between concurrent changes in dependent and independent variables across the three-time intervals. Y variables (EQ-5D and EQ-VAS) were set to the change scores for the dependent factors. X variables included all independent factors and background variables. Thus, the OPLS analyses examined co-variation between changes in variables rather than temporal prediction. A positive change score indicates improvement (e.g., higher change score in EQ-5D or EQ-VAS equates to better HRQoL), whereas a negative change score indicates worsening. To identify which X variables were most important for changes in Y variables, those with a variable influence on projection (VIP) value of at least 1.0 and a VIP value greater than the standard error were included in a second OPLS model with one predictive and one orthogonal component.

Third, multivariate models using OPLS-DA were employed to investigate whether background variables and baseline measurements of any independent factors could predict clinically significant improvement in the dependent factors (EQ-5D and EQ-VAS). Individuals with clinically improved values (change in EQ-5D ≥ 0.03 and EQ-VAS ≥ 10.5) were compared with those with no improvement or deteriorated values (change ≤ 0) for HRQoL at 3 months and 15 months. Like OPLS, VIP-selection was used to assess which X-variables were important for separation of the groups.

All multivariate models of HRQoL were assessed for quality using the goodness of fit (R²) and predictive ability (Q²). The significance of group separation was assessed using cross-validated analysis of variance (CV-ANOVA), where p < 0.05 was considered significant. The maximum value for R² and Q² is 1.0, symbolizing a perfect model. Scaled loadings (weights representing the variables) were used to interpret the association between X (independent factors) and Y (dependent factors) variables. In addition, permutation testing was performed for significant OPLS-DA model to further assess model validity.

Sample size and power calculations for the host RCT were based on the primary outcome, the NDI, and were conducted before data collection commenced; no corresponding calculations were undertaken for the secondary outcomes [10, 16]. A post hoc sample size calculation for HRQoL was performed using a one-sided test, 80% power, and the non-inferiority margins specified in the host trial [10]; the required sample size was estimated separately using the pooled standard deviations at 3 and 15 months:

At 3 months, 79 participants per group were required for EQ-5D and 53 per group for EQ-VAS. At 15 months, the corresponding requirements were 72 and 56 participants per group, respectively. Allowing for 20% attrition, the recruitment targets would have been 99 and 67 participants per group at 3 months, and 90 and 70 participants per group at 15 months, for EQ-5D and EQ-VAS, respectively. In the host trial [10] we recruited 70 participants per group; thus, the sample size was sufficient for EQ-VAS but below the estimated requirement for EQ-5D at both follow-ups.

## Results

A summary of fitted continuous-outcome OPLS and group separating OPLS-DA models is provided in Table 2. A total of 130 individuals completed the follow-ups. First, three OPLS-DA models were constructed to investigate whether included (independent and dependent) variables were different between NSE and NSEIT interventions. Second, three OPLS models were performed to investigate associations between changes in independent variables and changes in the dependent variables, EQ-5D and EQ-VAS. Lastly, four OPLS-DA models were performed to investigate whether baseline values of independent variables could predict clinically relevant improvement in EQ-5D or EQ-VAS at 3 months and 15 months follow-up.

**Table 2:** Summary of fitted OPLS and OPLS-DA models

OPLS-DA models – comparison interventions					
Purpose: Establish whether included variables can separate NSE and NSEIT					
Comparison	n participants NSE/NSEIT	R² / Q²	CV-ANOVA	Result	Figure no
NSE vs NSEIT Baseline vs 3 months	70/70	0,21 / −0,12	p = 1	No separation NSE and NSEIT	S4
NSE vs NSEIT Baseline vs 15 months		0,22 / 0,01	p = 1	No separation NSE and NSEIT	S5
NSE vs NSEIT 3 months vs 15 months		0,17 / −0,10	p = 1	No separation NSE and NSEIT	S6

OPLS-DA models – comparison clinically improved versus not improved						
Purpose: To distinguish clinically improved from non-improved participants based on baseline factors.						
Clinical improvement outcome	n (improved/-non- improved)	Initial model R² / Q²	R² / Q² after VIP-selection	Variables retained after VIP selection, n	CV-ANOVA	Figure no
EQ-5D at 3 months	62/64	0.18/ − 0.17	0.13 /0.02	12	p > 0.05	S7
EQ-5D at 15 months	58/53	0.31 / − 0.01	0.26 / 0.13	19	p < 0.01	6
EQ-VAS at 3 months	55/39	0.29 / − 0.16	0.14 / − 0.05	13	p > 0.05	S8
EQ-VAS at 15 months	46/37	0.25 / − 0.37	0.06 / − 0.12	8	p > 0.05	S9

OPLS models – continuous outcome EQ-5D and EQ-VAS						
Purpose: Find associations of improvements in independent variables and EQ-5D/EQ-VAS						
Comparison	n participants	Initial model R² / Q²	R² / Q² after VIP-selection	Variables retained after VIP selection, n	CV-ANOVA	Figure no
Baseline vs 3 months	140	0.28 / − 0.03	0.21 / 0.13	14	EQ-5D: p > 0.05 EQ-VAS: p < 0.001	2
Baseline vs 15 months		0.30 / 0.07	0.27 / 0.15	18	EQ-5D: p < 0.001 EQ-VAS: p < 0.05	4
3 vs 15 months		0.23 / − 0.16	0.20 / 0.06	18	EQ-5D: p < 0.05 EQ-VAS: p > 0.05	5

*R²*, goodness of fit, *Q²*, predictive ability, *VIP*, variable importance in projection, *CV-ANOVA*, cross-validated analysis of variance

The OPLS models included EQ-5D and EQ-VAS simultaneously as response variables. Clinically improved participants were defined as a change ≥0.03 for EQ-5D and ≥10.5 points for EQ-VAS

### Comparison NSE and NSEIT

Using the improvement (changes scores) of both independent and dependant variables, the models showed no apparent separation of NSEIT vs. NSE and were not significant (CV-ANOVA p = 1, S Figs. 4–6). This verifies the results from the main outcome RCT demonstrating no significant differences between the NSEIT and NSE [10] and allowed that all participants were treated as one group.

### Association between improvements in independent variables and HRQoL

**Fig. 2:**
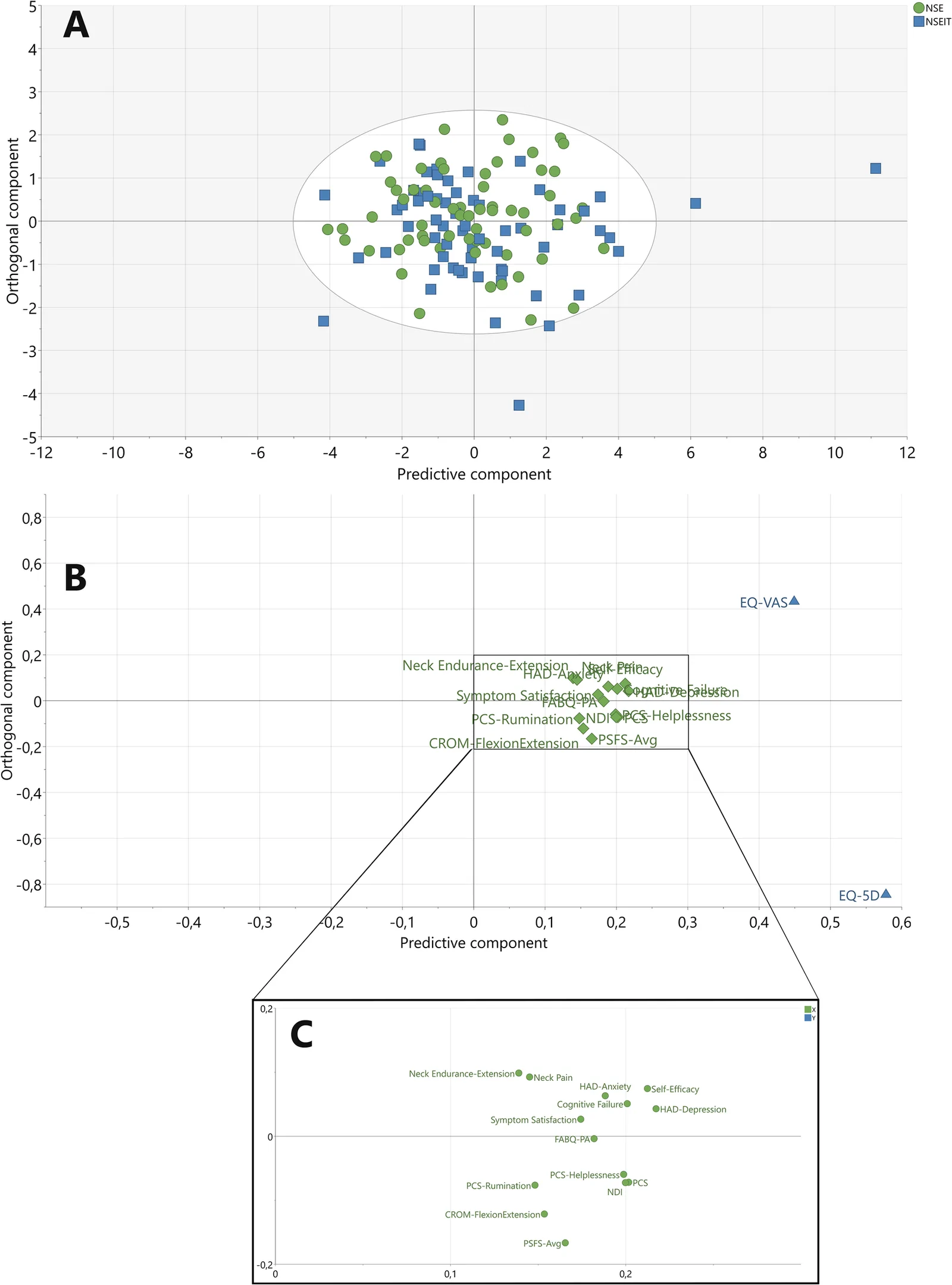
Multivariate modelling using orthogonal partial least square (OPLS) for change scores (where increasing values indicate improvement) from baseline to 3 months. **A**: Score plot of the participating patients. The colour corresponds to grouping in either NSE (green circles) or NSEIT (blue squares). **B**: Loading plot of analysed factors underlying the separation in the score plot. The x-axis depicts the predictive component most important for separating participants based on improvements in EQ-VAS and EQ-5D in both the NSEIT and NSE groups. R^2^ = 0.21, Q^2^ = 0.13, CV-ANOVA p < 0.001 for EQ-VAS and p > 0.05 for EQ-5D

**Fig. 3:**
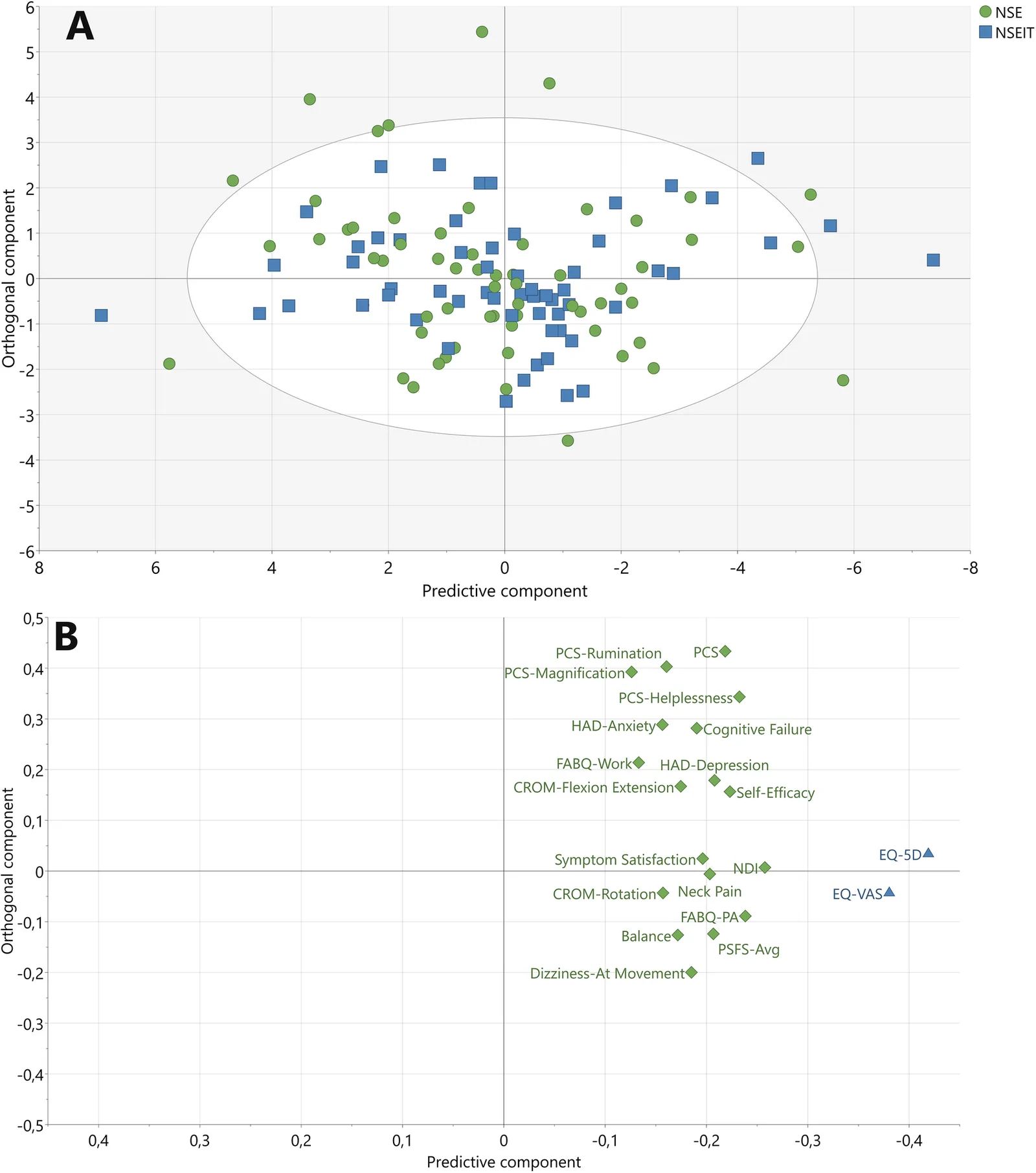
Multivariate modelling using orthogonal partial least square (OPLS) for change scores (where increasing negative values/longer stack indicate improvement) from baseline to 15 months. **A**: Score plot of the participating patients. The colour corresponds to grouping in either NSE (green circles) or NSEIT (blue squares). **B**: Loading plot of analysed factors underlying the separation in the score plot. The x-axis depicts the predictive component most important for separating participants based on improvements in EQ-VAS and EQ-5D in both the NSEIT and NSE groups. R^2^ = 0.27, Q^2^ = 0.15, CV-ANOVA p < 0.05 for EQ-VAS and p < 0.001 for EQ-5D

**Fig. 4:**
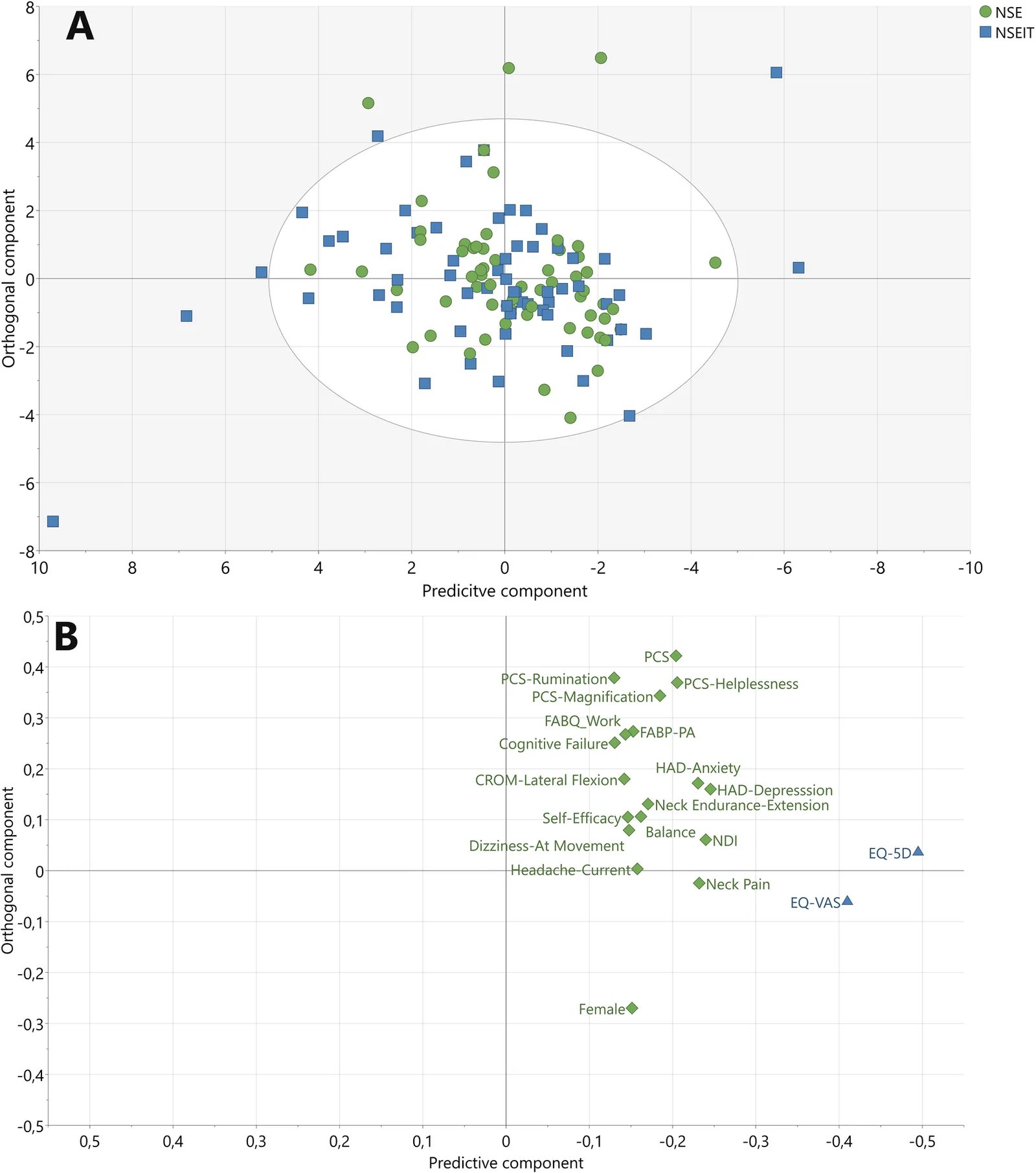
Multivariate modelling using orthogonal partial least square (OPLS) for change scores (where increasing values indicate improvement) from 3 to 15 months. **A**: Score plot of the participating patients. The colour corresponds to grouping in either NSE (green circles) or NSEIT (blue squares). **B**: Loading plot of analysed factors underlying the separation in the score plot. The x-axis depicts the predictive component most important for separating participants based on improvements in EQ-VAS and EQ-5D in both the NSEIT and NSE groups. R^2^ = 0.20, Q^2^ = 0.06, CV-ANOVA p > 0.05 for EQ-VAS and p < 0.05 for EQ-5D

**Fig. 5:**
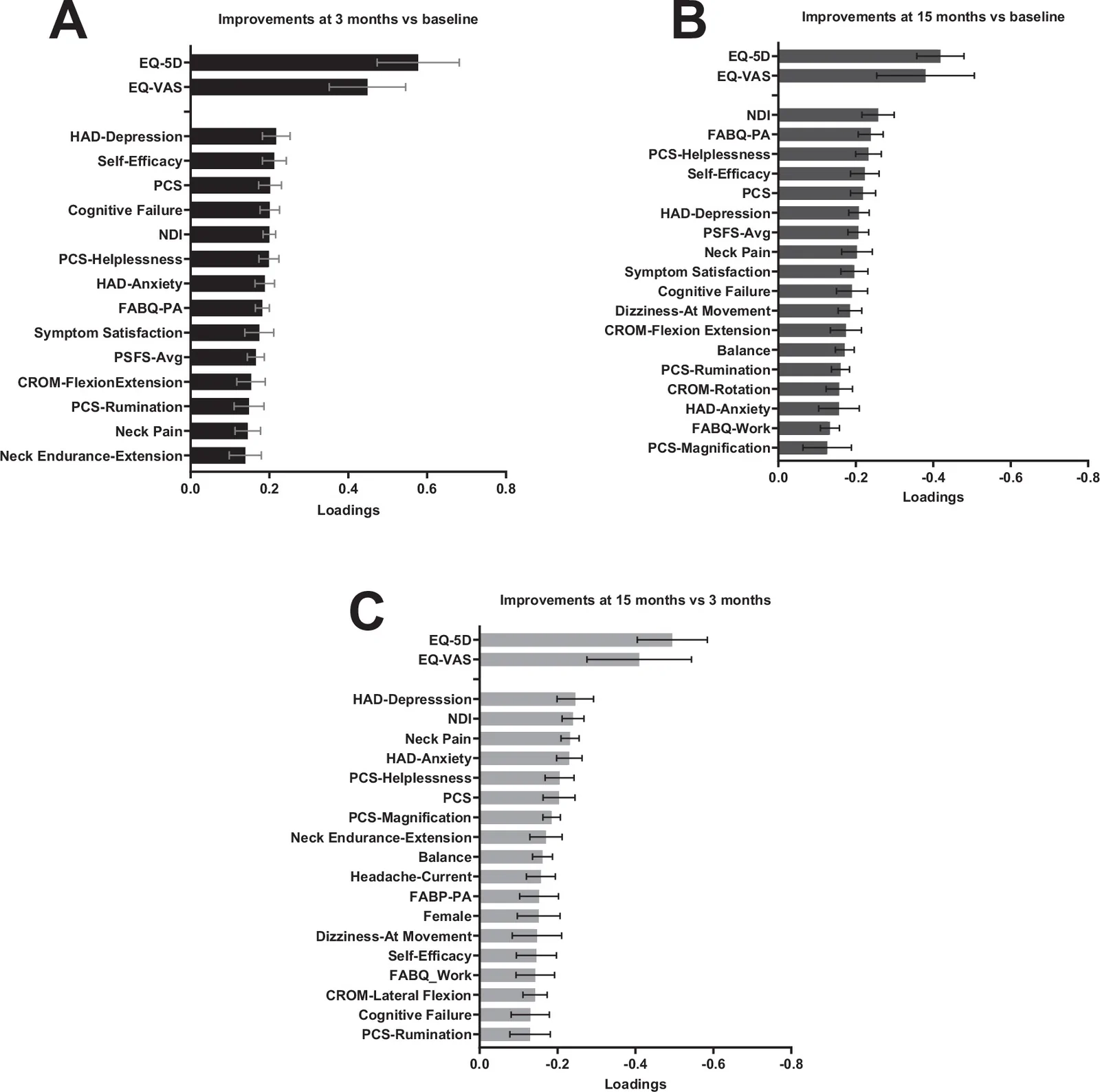
Loadings and their cross-validated standard error (cvSE, whiskers) for improvements of variables along the predictive x-axis obtained through OPLS after selecting variables with VIP > 1 and VIP > SE. **A**: Change score showing improvements between baseline and 3-month follow-up **B**: Change score showing improvements between baseline and 15-month follow-up. **C**: Change score showing improvements between 3- and 15-month follow-up. An increasing negative value/longer stack for the loadings indicates that it is more important in the models

OPLS models of improvement (change score) of independent variables in relation to HRQoL variables EQ-5D and EQ-VAS were modelled for all time point comparisons (Figs. 2, 3 and 4). The models showed low goodness of fit (R²) and weak predictive ability (Q²). CV-ANOVA was significant for both EQ-5D and EQ-VAS for the baseline-to-15-month interval, whereas only one of the two outcomes was significant for each of the other intervals. The models resulted in 14, 18 and 17 independent variables associated with the outcomes (Fig. 5). The three variables with the largest loading (importance) in each model were HAD-Depression, Self-efficacy and PCS in baseline vs 3 months (Fig. 5A), NDI, FABQ-PA, and PCS-Helplessness in baseline vs 15 months (Fig. 5B) and HAD-Depression, NDI, and neck pain intensity in 3 months vs 15 months (Fig. 5C).

### Differentiation of clinical improvement in HRQoL based on baseline factors

**Fig. 6:**
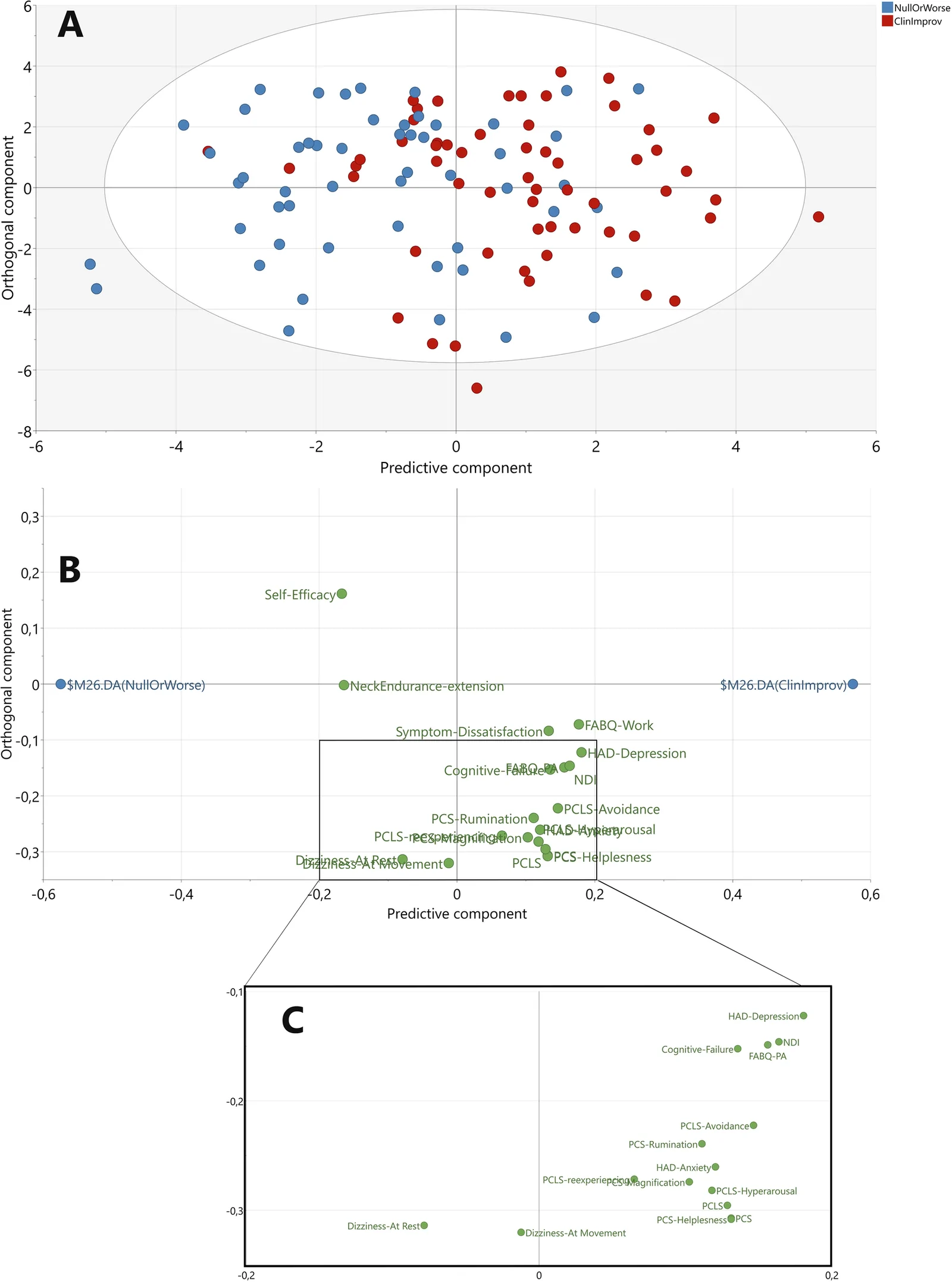
OPLS-DA model of clinically improved individuals (≥0.03 increment in EQ-5D) compared to individuals with non-effect/worse EQ-5D (≤0) at 15-month follow-up, based on the baseline variables. **A**: Score plot of the participating patients. The colour corresponds to grouping in null or worse effect (blue inverted triangles) or clinically improved (red pentagons). **B**: Loading plot of analysed factors underlying the separation in the score plot. **C**: Inlay of lower part of loading plot for enhanced visibility. The x-axis depicts the predictive component most important for separating groups, while the y-axis represents the orthogonal component showing in-group variation, R^2^ = 0.26, Q^2^ = 0.13, CV-ANOVA p < 0.01

The ability to differentiate clinically improved in HRQoL and non-improved participants based on baseline values (of independent factors) was investigated using OPLS-DA. Patients who had a clinical improvement in EQ-5D (defined as a 0.03 increase) or EQ-VAS (defined as a 10.5 increment) [18] were compared to patients with no or worse values at 3 months and 15 months (Fig. 6).

No significant models were obtained for EQ-5D at 3 months, EQ-VAS at 3 months, or EQ-VAS at 15 months (Figs. S7–S9).

However, for clinical improvement of EQ-5D at 15 months a significant model could be built after VIP-selection containing 19 variables (Fig. 4). This model showed low goodness of fit and predictability (R^2^ = 0.26 and Q^2^ = 0.13) but it was significant (CV-ANOVA p < 0.01). Variables associated with clinical improvement were higher baseline values for HAD-Depression, FABQ-Work, NDI, FABQ-PA, PTSD-avoidance, CFQ, Symptom (Dis)satisfaction, PCS-Helplesness, PCS total score, PTSD total score, HAD-Anxiety, PTSD-Hyperarousal, PCS-Rumination, PCS-Magnification and PTSD-Reexperiencing. Variables negatively associated with clinical improvement were high Dizziness during activity and at rest, high SES, and high NME-extension. Permutation testing of the significant OPLS-DA model showed higher R² and Q² values for the original model than for permuted models, and the Q² intercept was negative, suggesting that the observed class separation was unlikely to be explained by chance alone (Fig. S10). A sensitivity analysis of the baseline values regarding clinical improvement of EQ-5D at 15 months compared to no or worse EQ-5D was done adding EQ-5D baseline values as a predictor. This showed that lower EQ-5D values at baseline were associated with clinical improvement at 15months (Fig. S11).

## Discussion

This is the first study to examine factors associated with HRQoL outcomes following internet-supported exercise for chronic WAD. No significant differences were found between NSE and NSEIT, consistent with the RCT’s primary [10] and work-ability outcomes [9]. However, as the secondary analyses of HRQoL were exploratory and the analyses of EQ-5D were underpowered, the possibility that this finding was affected by a type II error cannot be excluded.

### Clinically relevant improvement in HRQoL

Clinically relevant improvement in EQ-5D-3L at 15 months was associated with greater baseline psychosocial burden (HAD, FABQ, PTSD, CFQ, PCS and symptom dissatisfaction), higher NDI, and lower dizziness, SES and NME extension. Participants with poorer baseline HRQoL may have had greater scope for improvement, as supported by the sensitivity analysis. The association between adverse psychosocial factors and improvement was unexpected because these factors have predicted poorer outcomes after cervical disc surgery, in chronic pain and after WAD [35–38]. Carefully progressed low-load neck-specific exercise combined with reassuring information may have increased confidence to exercise and improved both psychosocial outcomes and HRQoL. Psychosocial improvements also had among the highest OPLS loadings at both follow-ups. Although cognitive behavioural therapy has shown small or no clinically meaningful effects in chronic neck pain [39], the combined exercise and information in NSEIT/NSE may have been beneficial. Differences from earlier findings may reflect variation in populations, variables and analytical methods; unlike most previous studies, the present analyses used change scores, while the findings accord with those of Landén Ludvigsson et al. [11]. Cognitive failure has not previously been examined as a predictive factor in chronic WAD and warrants further study. Lower baseline dizziness characterised participants who improved, consistent with evidence linking dizziness to poorer quality of life and depression [36, 40, 41]; patients with dizziness or greater psychosocial burden may therefore need additional support. Higher baseline SES and dorsal NME were associated with less improvement, possibly because participants with higher initial scores were closer to healthy reference levels and therefore had less scope for improvement when change scores were used in the analyses. SES has been related to quality of life, health, pain and disability [42–44], and NME to pain, disability and dizziness [45, 46]. Neck-specific exercise has also been associated with reduced pain and disability in chronic WAD [47].

No significant 3-month model was obtained, possibly indicating that continued exercise is important. However, only one of four OPLS-DA models was significant. The significant EQ-5D but non-significant EQ-VAS model at 15 months may reflect differences between a multidomain measure and a single global rating. Given the inconsistent model significance and weak predictive performance, the findings should be interpreted cautiously as exploratory and hypothesis-generating.

### Association of improvements in independent variables with improvements in HRQoL at 3- and/or 15-month follow-up

Alongside psychosocial variables, improvements in NDI and pain intensity were among the principal factors associated with improved HRQoL at 3 and/or 15 months. NDI has previously been associated with work ability in chronic WAD [48, 49], perceived recovery in WAD and other pain conditions, and outcomes after spinal surgery [35–37, 50–54]; disability and insurance claims have also been strongly associated with global perceived recovery [55]. Its importance for HRQoL at 15 months was therefore expected. Conversely, baseline pain intensity and NDI were not influential in the short-term model, in which psychosocial factors, AROM and NME extension predominated. Previous studies have associated higher pain intensity with worse outcomes after neck surgery, HRQoL improvement following neck-specific exercise, and poorer prognosis [11, 35, 56]. Cognitive failure was important in all three HRQoL models despite the exclusion of participants with traumatic brain injury.

Objective measures, including NME, AROM and balance, were also associated with HRQoL at 3 and/or 15 months. AROM has been linked to pain and disability after neck surgery [44] and persistent dizziness in chronic WAD [46], although its prognostic importance for WAD chronicity appears limited [56]. Balance has previously predicted dizziness [46]; here, improved single-leg balance with eyes closed was associated with improved HRQoL from baseline and 3 months to 15 months. Objective measures have rarely been examined as predictors of HRQoL.

Adherence was not influential, possibly because most participants adhered to the programme. Background characteristics were also unimportant, unlike findings after cervical disc surgery for sex and smoking [45], but consistent with exercise studies in chronic WAD [11, 46–48]. Although poorer finances have been associated with work ability in chronic WAD [49], relevant factors may vary by outcome.

The findings also accord with wider WAD evidence identifying psychosocial distress, pain, disability and recovery expectations as prognostic factors for long-term outcomes [4, 38, 50, 51, 54, 56]. Although these studies primarily examined pain, disability or perceived recovery, they reinforce the importance of psychological and behavioural factors in WAD.

Landén Ludvigsson et al. [11] is the only previous study of factors associated with HRQoL improvement after neck-specific exercise in chronic WAD. Its population and programme were similar, but it used a 6-month follow-up, no internet support and two-dimensional regression (Table S12).

### Strengths and limitations

To our knowledge, this is the first study to examine factors associated with EQ-5D or EQ-VAS outcomes after internet-supported neck-specific exercise in chronic WAD.

Delivery by 57 physiotherapists across a wide geographical area limited intervention control but strengthened clinical generalisability across levels of experience.

OPLS can simultaneously model numerous correlated variables, accommodate multicollinearity and missing data, and give scaled, mean-centred variables equal opportunity to influence the model. It can also identify participant and variable profiles, correlation patterns and the most influential variables, offering advantages over conventional two-dimensional regression.

The OPLS and OPLS-DA models nevertheless had several limitations. Most showed weak fit and predictive ability, suggesting that unmeasured factors, including general health, family circumstances and leisure activities, may influence HRQoL. EQ-5D-3L may also be insufficiently responsive to neck-related change compared with EQ-5D-5L, and ceiling effects or limited sensitivity may partly explain the modest model performance. VIP-based variable selection may introduce optimistic bias and overfitting, although the reduction in R² and increase in Q² after selection were consistent with removal of less informative variables. The limited sample size relative to the number of variables may also have caused model instability. Permutation testing indicated that separation between participants with and without clinically relevant EQ-5D improvement at 15 months was unlikely to be due to chance. Nonetheless, the modest explanatory and predictive performance requires cautious interpretation and independent validation; the models should be regarded as exploratory and hypothesis-generating rather than suitable for individual prediction.

A further limitation was that the a priori statistical power calculation for the host RCT was based on the primary outcome, the NDI [10], and did not account for secondary outcomes, including HRQoL. Post hoc sample size analyses indicated that the sample size was adequate for EQ-VAS but insufficiently powered for EQ-5D.

As thresholds for clinically relevant change in EQ-5D and EQ-VAS have not been established for neck disorders, values derived from a low-back-pain population were applied [18].

The findings may be generalisable to individuals meeting the strict RCT criteria, but not to all people with WAD. One-third of participants were men despite sex-stratified randomisation and equal opportunity to participate; this distribution resembles that reported by Landén Ludvigsson et al. [11] and may reflect the greater prevalence of whiplash injuries among women [57]. Requiring sufficient Swedish proficiency to understand the intervention and questionnaires further limited generalisability, although ethnicity was not an exclusion criterion.

## Conclusions

Clinical improvement in EQ-5D-3L was associated with worse baseline psychosocial variables (HAD, FABQ, PTSD, CFQ, PCS, Symptom satisfaction) as well as lower dizziness, SES and NME-extension. Furthermore, it is noteworthy that improvements in psychosocial factors, and pain intensity, NDI, NME-extension, AROM, dizziness, balance, headache, and cognition, were significantly associated with improvements in HRQoL at 3- and/or 15-month follow-ups. Given the methodological limitations and rather weak models, these exploratory findings should be interpreted with caution and require confirmation in independent cohorts before conclusions can be drawn regarding the influence of baseline psychosocial distress on outcomes from neck-specific exercise.

## Supplementary information

S1. CONSORT 2010 checklist

S2. SPIRIT checklist

S3. Supplementary table

S4 NSE NSEIT BL vs 3M

S5 NSE NSEIT BL vs 15M

S6 NSE NSEIT 3M vs 15M

S7 EQ5D Bl to 3M

S8 EQVAS Bl to 3M

S9 EQVAS Bl to 15M

S10 Permutation analysis

S11 Sensitivity analysis

S12 Supplementary table Comparison with Landén Ludvigsson et al. table

Supplementary information

## Data Availability

The data sets generated during this study are available from the corresponding author upon reasonable request. Our data cannot be made publicly available for both ethical and legal reasons as public availability would compromise patient privacy and information about their health. Coded data will be received upon reasonable request and after ethical permission. For a non-author point of contact: registrator@hmv.liu.se

## List of Abbreviations

WAD: whiplash-associated disorders
HRQoL: health-related quality of life
RCT: Randomized controlled trial
NSE: neck-specific exercise programme followed twice a week for 3 months at a physiotherapy clinic
NSEIT: neck-specific exercise programme delivered via 4 physiotherapy visits combined with an internet-based programme
VAS: Visual Analog Scale
NDI: Neck Disability Index
Y-variables: Dependent/response variables
X-variables: Independent/explanatory factors
EQ-5D-3L: Euroqol five dimensions
EQ-VAS: Euroqol vertical Visual Analog Scale
PTSD: Post Traumatic Stress Disorder
PCL-S: Post Traumatic Stress Disorder Checklist
QTF: Quebec Task Force classification
PSFS: Patient-Specific Functional Scale
FABQ: Fear-Avoidance Beliefs Questionnaire
PA: Physical Activity Subscale of FABQ
HAD: Hospital Anxiety and Depression Scale
PCS: Pain Catastrophizing Scale
SES: Self-Efficacy Scale
CFQ: Cognitive Failures Questionnaire
NME: neck muscle endurance
CROM: Cervical range of motion instrument
CCFT: cranio-cervical flexion test (CCFT),
SOLEC: balance test standing on one leg with eyes closed
OPLS: Orthogonal partial least squares
OPLS-DA: OPLS discriminant analysis
R²: Goodness of fit
Q²: Predictive ability
CV-ANOVA: cross-validated analysis of variance
