# Supplementary material for: Factors associated with 1-year health-related quality of life after neck-specific exercise in chronic whiplash-associated disorders: an exploratory secondary analysis of a randomized controlled trial
Source: BMC Musculoskelet Disord. 2026 Oct 13;27:871. doi: 10.1186/s12891-026-10581-x (PMC13670587; doi:10.1186/s12891-026-10581-x)
Supplement: Supplementary file 1 [file 12891_2026_10581_MOESM10_ESM.pdf]

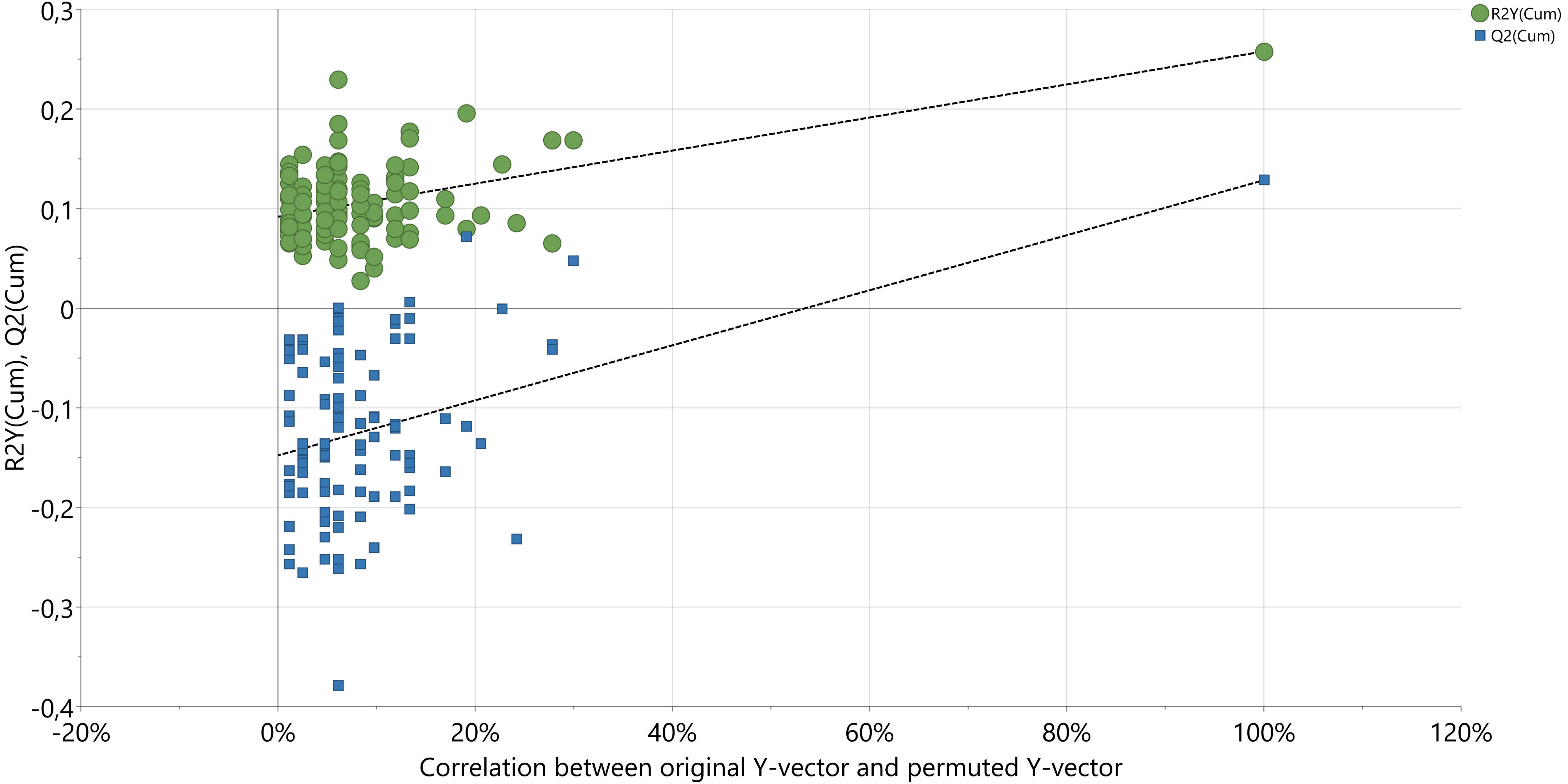
R2Y(Cum)
Q2(Cum)
R2Y(Cum), Q2(Cum)
0,3
0,2
0,1
0
-0,1
-0,2
-0,3
-0,4
-20%
0%
20%
40%
60%
80%
100%
120%
Correlation between original Y-vector and permuted Y-vector
