# Supplementary material for: Factors associated with 1-year health-related quality of life after neck-specific exercise in chronic whiplash-associated disorders: an exploratory secondary analysis of a randomized controlled trial
Source: BMC Musculoskelet Disord. 2026 Oct 13;27:871. doi: 10.1186/s12891-026-10581-x (PMC13670587; doi:10.1186/s12891-026-10581-x)
Supplement: Supplementary file 2 [file 12891_2026_10581_MOESM11_ESM.pdf]

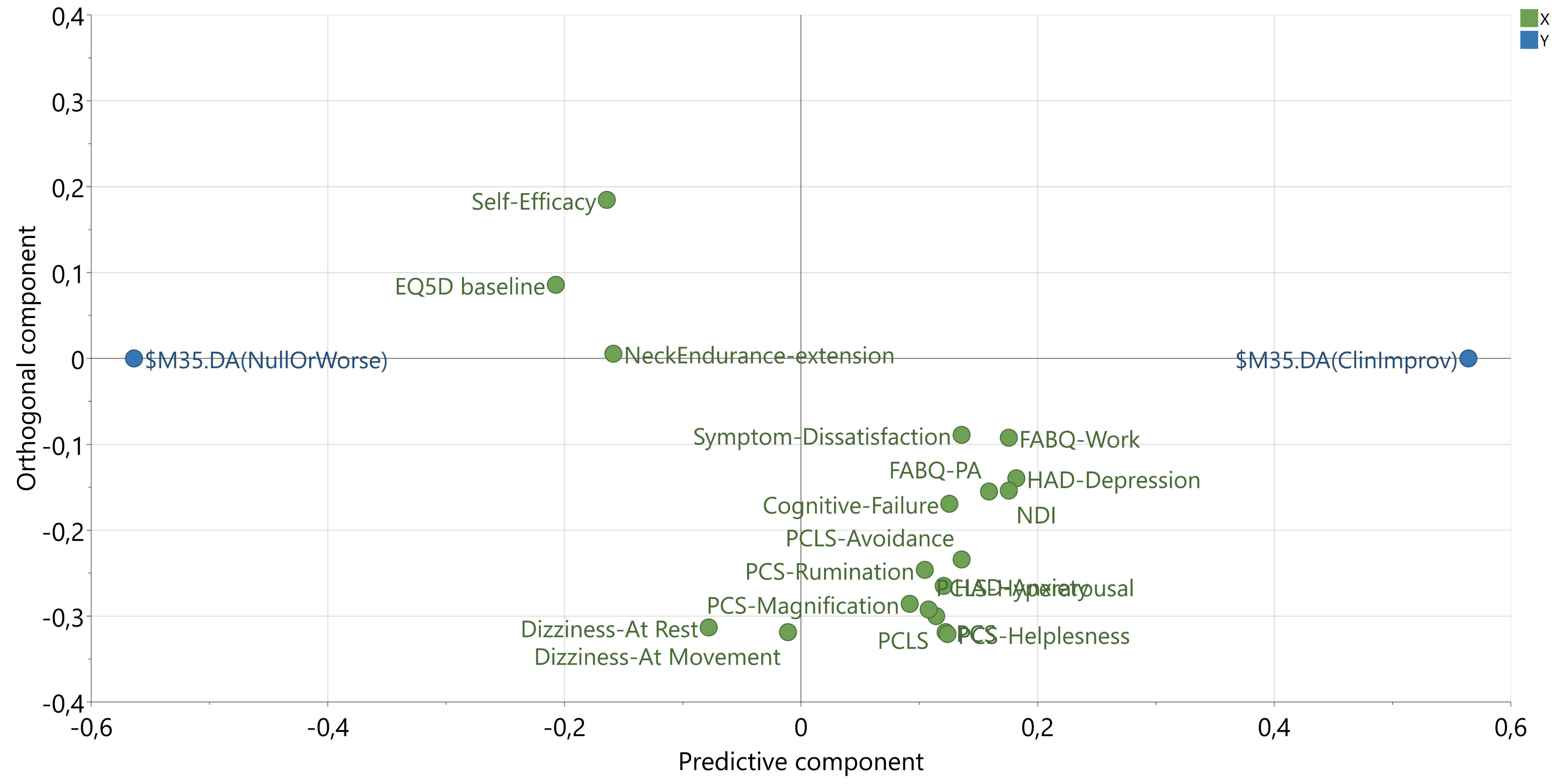

X
Y
0,4
0,3
0,2
0,1
0
-0,1
-0,2
-0,3
-0,4
Orthogonal component
-0,6
-0,4
-0,2
0
0,2
0,4
0,6
Predictive component
Self-Efficacy
EQ5D baseline
$M35.DA(NullOrWorse)
NeckEndurance-extension
$M35.DA(ClinImprov)
Symptom-Dissatisfaction
FABQ-Work
FABQ-PA
HAD-Depression
Cognitive-Failure
NDI
PCLS-Avoidance
PCS-Rumination
PCLS-Hyperarousal
HAD-Anxiety
PCS-Magnification
Dizziness-At Rest
Dizziness-At Movement
PCLS
PCS
PCS-Helplesness
