# Supplementary material for: Factors associated with 1-year health-related quality of life after neck-specific exercise in chronic whiplash-associated disorders: an exploratory secondary analysis of a randomized controlled trial
Source: BMC Musculoskelet Disord. 2026 Oct 13;27:871. doi: 10.1186/s12891-026-10581-x (PMC13670587; doi:10.1186/s12891-026-10581-x)
Supplement: Supplementary file 3 [file 12891_2026_10581_MOESM12_ESM.pdf]

Table, S12. Comparison of health-related quality of life outcomes and associated factors in the present study and the previous randomized trial by Landén Ludvigsson et al. 2019 (reference 11). In both studies the intervention period was 3 months.

Domain	Present study	Landén Ludvigsson et al. 2019
Study design	Secondary analysis of a randomized controlled multicentre trial comparing neck-specific exercise with internet support (NSEIT) with neck-specific exercise at a physiotherapy clinic (NSE). The manuscript used OPLS and OPLS-DA models to investigate factors associated with changes in EQ-5D-3L and EQ VAS at 3- and 15-months follow-up.	Secondary analysis of a multicentre randomized clinical trial evaluating three exercise approaches regarding EQ-5D-3L and EQ VAS and factors associated with its improvement at 6 months follow-up using multivariate linear regression model. The exercise approaches were prescribed physical activity (PPA, general physical activity), neck-specific exercise (NSE), and neck-specific exercise with a behavioural approach (NSEB, as NSE but with additional cognitive behavioural component).
Participants	Participants with chronic WAD grades II–III were randomized to NSEIT or NSE (n=140).	Participants with chronic WAD grades II–III were included; the study comprised n = 216 participants randomized to PPA, NSE, or NSEB.
Intervention comparison	NSEIT was compared with NSE. No significant separation between NSEIT and NSE was found in the OPLS-DA models for change scores, supporting that the two delivery formats could be analysed together in the HRQoL models.	NSE and NSEB were compared with PPA. NSE/NSEB resulted in better outcomes than PPA for EQ VAS and SF-36 physical component summary, but not for EQ-5D. There were no significant differences between NSE and NSEB.
HRQoL outcomes	EQ-5D-3L and EQ VAS were used as dependent HRQoL outcomes.	EQ-5D-3L and EQ VAS were used as dependent HRQoL outcomes.
Follow-up focus	The analysis focused on changes from baseline to 3 months, baseline to 15 months, and 3 to 15 months.	The study analysed outcomes across baseline, 3, 6, and 12 months, with multivariate regression analyses for baseline and 6-month HRQoL.
Main HRQoL treatment effect	No significant differences were found between NSEIT and NSE in the comparison models, indicating comparable effects of the internet-supported and clinic-based	Neck-specific exercise, particularly NSEB, was reported to have a more positive impact on HRQoL than PPA in chronic WAD grades II–III.

	programmes on HRQoL-related change patterns.	
EQ-5D findings	OPLS and OPLS-DA models. A significant prediction model for clinically relevant improvement in EQ-5D at 15 months was reported, with $R^2 = 0.25$, $Q^2 = 0.13$ and CV-ANOVA $p < 0.01$.	NSE/NSEB did not result in a higher EQ-5D score than PPA, although improvement over time in EQ-5D/EQ VAS was reported for the NSEB group.
EQ VAS findings	OPLS models for improvement in HRQoL showed significant models for EQ VAS in the baseline-to-3-month model and the baseline-to-15-month model.	NSE/NSEB resulted in better EQ VAS outcomes than PPA, with $p < 0.01$.
Important factors associated with short-term HRQoL improvement	For baseline to 3 months, the model had $R^2 = 0.20$ and $Q^2 = 0.12$; the three most important related factors were improvement in HAD Anxiety, HAD Depression, and FABQ-PA.	The study reported that factors associated with baseline HRQoL and HRQoL improvement were multidimensional and included both neck-related and psychosocial factors. Specific examples given were depression and work ability.
Important factors associated with longer-term HRQoL improvement	For baseline to 15 months, the model had $R^2 = 0.27$ and $Q^2 = 0.15$; the three most important related factors were improvement in NDI, FABQ-PA, and PCS helplessness.	In the study, factors associated with baseline HRQoL and change to 6 months in HRQoL had R^2 values of 0.38–0.59 and included both neck-related and psychosocial factors.
Factors associated with later change from 3 to 15 months	The 3-to-15-month model had $R^2 = 0.20$ and $Q^2 = 0.06$; the three most important related factors were improvement in HAD Depression, NDI, and neck pain intensity.	The study focused on baseline and 6-month regression models and does not report a directly comparable 3-to-15-month model.
Baseline predictors of clinically relevant improvement	Worse baseline psychosocial variables were associated with clinically relevant improvement in EQ-5D at 15 months. Variables associated with clinical improvement included higher baseline HAD Depression, FABQ Work, FABQ-PA, PTSD avoidance, CFQ, PCS total, PCS helplessness, symptom dissatisfaction, PTSD	The study reported that factors associated with HRQoL and HRQoL improvement were multidimensional, including psychosocial factors such as depression and work ability, but a detailed baseline predictor list as is missaing.

	total, HAD Anxiety, PTSD hyperarousal, PCS rumination, PCS magnification, and PTSD re-experiencing.	
Factors negatively associated with clinical improvement	Factors negatively associated with clinically relevant improvement in EQ-5D at 15 months were high dizziness during activity, high dizziness at rest, high self-efficacy, and high dorsal neck muscle endurance at baseline.	The study does not specify negatively associated baseline factors.
Overall interpretation	The study extends previous work by showing that improvements in psychological factors, pain, disability, cognition, dizziness/balance, and objective physical measures were related to HRQoL improvement after NSEIT or NSE, with no clear difference between delivery formats.	The study showed that neck-specific exercise, especially with a behavioural approach, may improve HRQoL more than prescribed physical activity, and that HRQoL improvement is multidimensional.
Contribution to the evidence base	The study adds evidence on internet-supported delivery of neck-specific exercise and multivariate modelling of EQ-5D-3L and EQ VAS outcomes.	The study provides earlier evidence that neck-specific exercise with or without behavioural support has a more favourable HRQoL profile than prescribed physical activity in chronic WAD.

Abbreviations: WAD, whiplash-associated disorders; NSEIT, neck-specific exercise with internet support; NSE, neck-specific exercise at a physiotherapy clinic; NSEB, neck-specific exercise with a behavioural approach; PPA, physical activity prescription; HRQoL, health-related quality of life; EQ-5D-3L, EuroQol five-dimension three-level questionnaire; EQ VAS, EuroQol Visual Analogue Scale; SF-36v2, Short Form Health Survey version 2; PCS, Pain Catastrophizing Scale; FABQ-PA, Fear-Avoidance Beliefs Questionnaire Physical Activity subscale; FABQ-W, Fear-Avoidance Beliefs Questionnaire Work subscale; HAD, Hospital Anxiety and Depression Scale; NDI, Neck Disability Index; CFQ, Cognitive Failures Questionnaire; PTSD, post-traumatic stress disorder; OPLS, orthogonal partial least squares; OPLS-DA, orthogonal partial least squares discriminant analysis.
