# Supplementary material for: Factors associated with 1-year health-related quality of life after neck-specific exercise in chronic whiplash-associated disorders: an exploratory secondary analysis of a randomized controlled trial
Source: BMC Musculoskelet Disord. 2026 Oct 13;27:871. doi: 10.1186/s12891-026-10581-x (PMC13670587; doi:10.1186/s12891-026-10581-x)
Supplement: Supplementary file 6 [file 12891_2026_10581_MOESM3_ESM.pdf]

Table S3. Missing data per timepoint expressed as %.

Missing data (%)	Baseline	3 months	15 months
EQ-5D	0	9	21
EQ-VAS	0	11	21
Age	0	NA	NA
Sex	0	NA	NA
Smoking	0	NA	NA
Education level	1	NA	NA
Months since injury	0	NA	NA
PCLS	44	NA	NA
PCLS-reexperiencing	44	NA	NA
PCLS-Avoidance	44	NA	NA
PCLS-Hyperarousal	44	NA	NA
WAD-grade	0	NA	NA
Expectation recovery	0	NA	NA
Training compliance	NA	11	NA
Current neck pain	0	14	14
Dizziness at rest	1	15	29
Dizziness at movement	1	14	29
Unsteadiness	1	14	29
Current headache	1	15	29
NDI	0	9	14
PSFS-avg	0	14	29
Symptom satisfaction	0	11	21
Exercise level	0	11	21
FABQ-PA	1	11	21
FABQ-work	1	11	21
HAD-anxiety	0	11	21
HAD-depression	0	11	21
PCS-Rumination	0	12	21
PCS-Magnification	0	12	21
PCS-Helplessness	0	12	21
SES	1	11	21
CFQ	1	11	21
Hand strength_Right	0	15	29
Hand strength_Left	1	14	29
Neck Endurance_ventral	0	15	29
Neck Endurance_dorsal	1	16	33
CROM_flexion_extension	0	14	29
CROM_lateral_flexion	0	14	29
CROM_rotation	0	14	29
Stabilizer	4	16	29
SOLEC	1	14	29
