# Supplementary material for: Factors associated with 1-year health-related quality of life after neck-specific exercise in chronic whiplash-associated disorders: an exploratory secondary analysis of a randomized controlled trial
Source: BMC Musculoskelet Disord. 2026 Oct 13;27:871. doi: 10.1186/s12891-026-10581-x (PMC13670587; doi:10.1186/s12891-026-10581-x)
Supplement: Supplementary file 7 [file 12891_2026_10581_MOESM4_ESM.pdf]

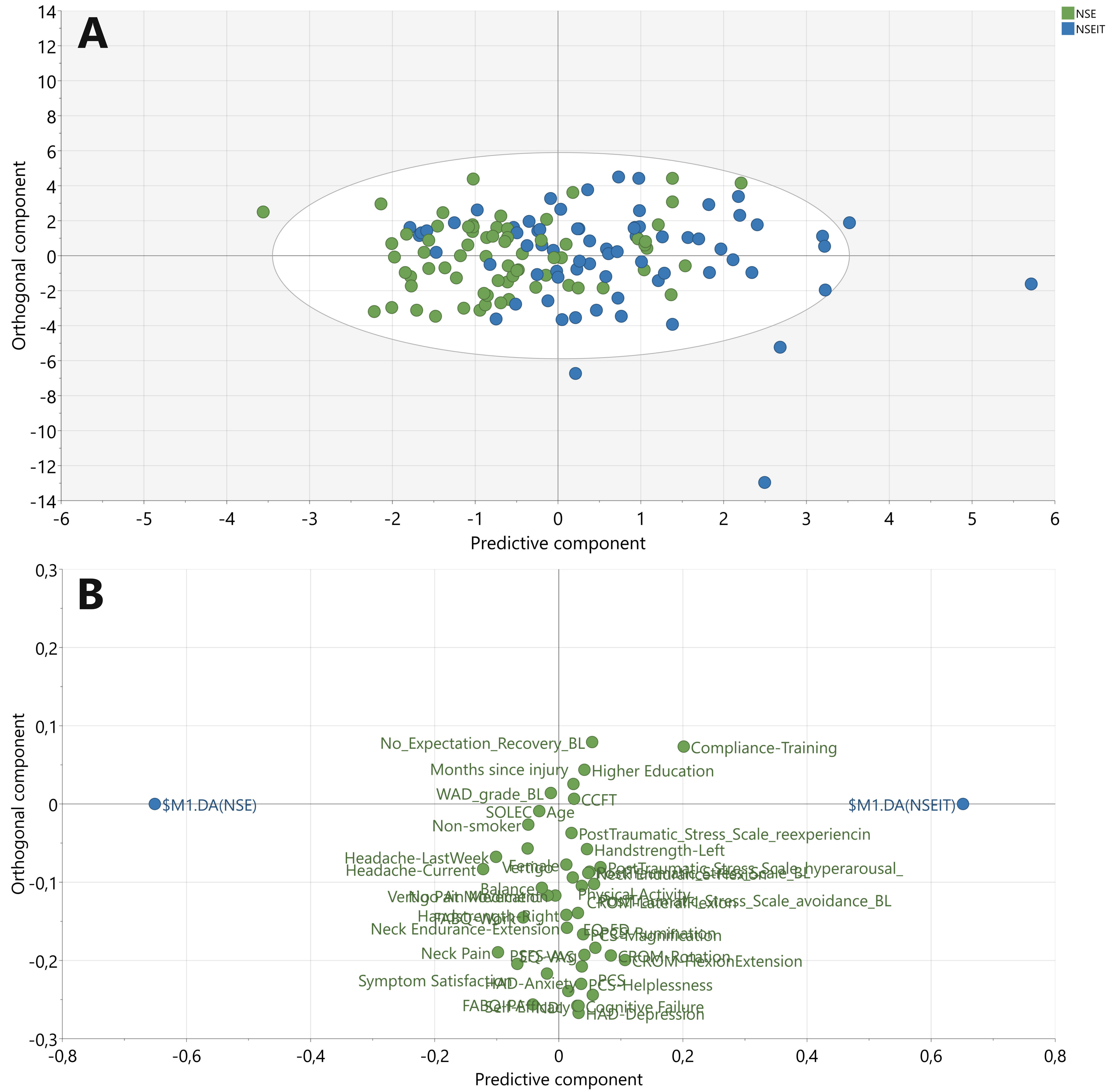

A
NSE
NSEIT
Orthogonal component
Predictive component
14
12
10
8
6
4
2
0
-2
-4
-6
-8
-10
-12
-14
-6
-5
-4
-3
-2
-1
0
1
2
3
4
5
6
B
0,3
0,2
0,1
0
-0,1
-0,2
-0,3
-0,8
-0,6
-0,4
-0,2
0
0,2
0,4
0,6
0,8
$M1.DA(NSE)
$M1.DA(NSEIT)
No_Expectation_Recovery_BL
Compliance-Training
Months since injury
Higher Education
WAD_grade_BL
CCFT
SOLEC
Age
Non-smoker
PostTraumatic_Stress_Scale_reexperiencin
Handstrength-Left
Headache-LastWeek
Headache-Current
Vertigo
Female
PostTraumatic_Stress_Scale_hyperarousal_
Balance
Physical Activity
PostTraumatic_Stress_Scale_avoidance_BL
CROM-LateralFlexion
Handstrength-Right
FABQ-Work
Neck Endurance-Extension
EQ-5D
PCS-Rumination
PCS-Magnification
Neck Pain
SES
VAS
CROM-Rotation
CROM-FlexionExtension
Symptom Satisfaction
HAD-Anxiety
PCS
PCS-Helplessness
Self-Efficacy
Cognitive Failure
HAD-Depression
