# Supplementary material for: Factors associated with 1-year health-related quality of life after neck-specific exercise in chronic whiplash-associated disorders: an exploratory secondary analysis of a randomized controlled trial
Source: BMC Musculoskelet Disord. 2026 Oct 13;27:871. doi: 10.1186/s12891-026-10581-x (PMC13670587; doi:10.1186/s12891-026-10581-x)
Supplement: Supplementary file 8 [file 12891_2026_10581_MOESM5_ESM.pdf]

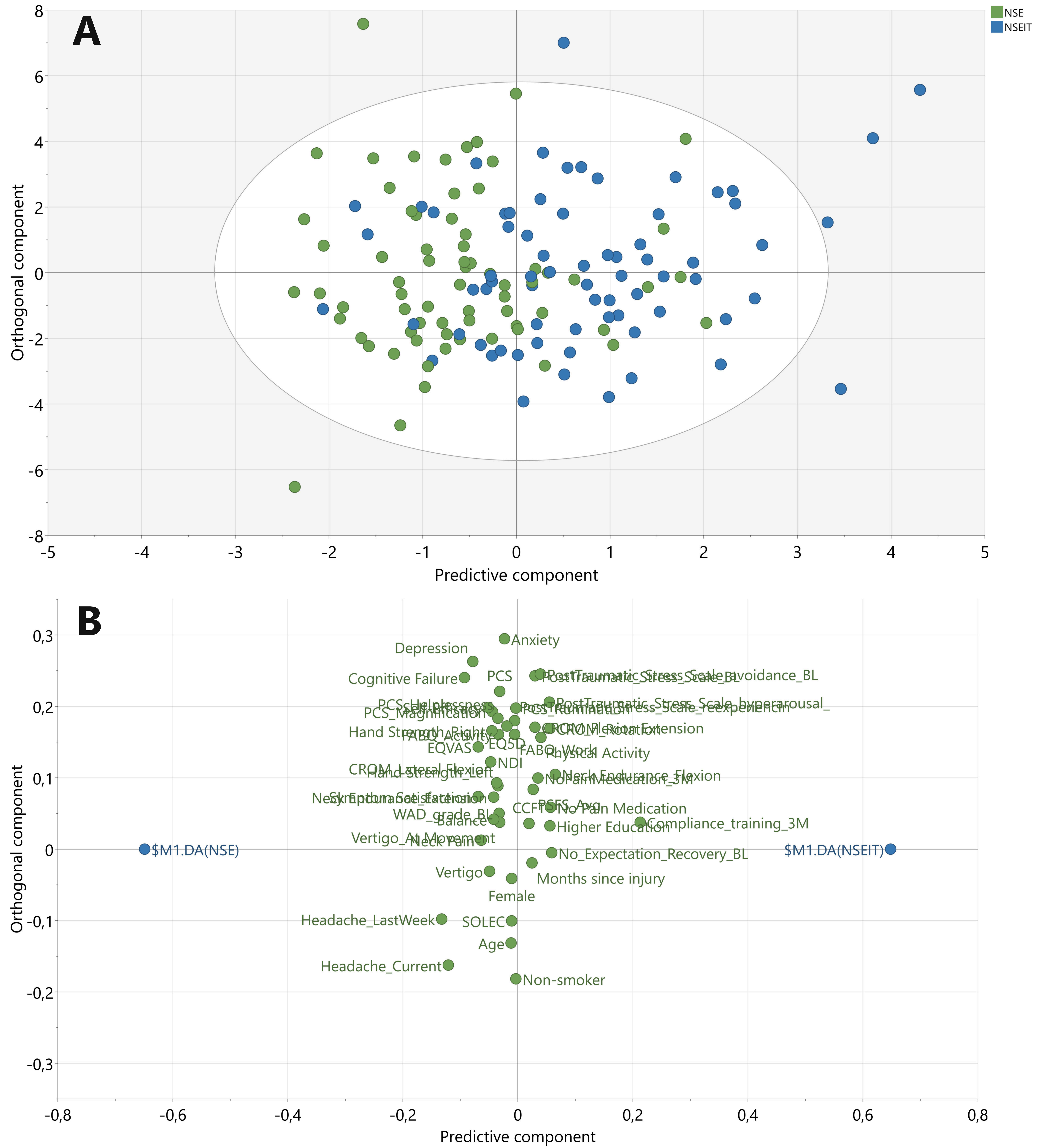

A
NSE
NSEIT
Orthogonal component
Predictive component
B
Anxiety
Depression
Cognitive Failure
PCS
NDI
EQVAS
EQ5D
Hand Strength_Left
CROM_Lateral_Flexion
WAD_grade_BL
Balance
Physical Activity
FABQ_Work
CCFT
PSFS_Avg
No Pain Medication
Higher Education
Compliance_training_3M
Neck Endurance_Flexion
$M1.DA(NSE)
$M1.DA(NSEIT)
Neck Pain
No_Expectation_Recovery_BL
Vertigo
Months since injury
Female
Headache_LastWeek
SOLEC
Age
Headache_Current
Non-smoker
Orthogonal component
Predictive component
