# Supplementary material for: Factors associated with 1-year health-related quality of life after neck-specific exercise in chronic whiplash-associated disorders: an exploratory secondary analysis of a randomized controlled trial
Source: BMC Musculoskelet Disord. 2026 Oct 13;27:871. doi: 10.1186/s12891-026-10581-x (PMC13670587; doi:10.1186/s12891-026-10581-x)
Supplement: Supplementary file 9 [file 12891_2026_10581_MOESM6_ESM.pdf]

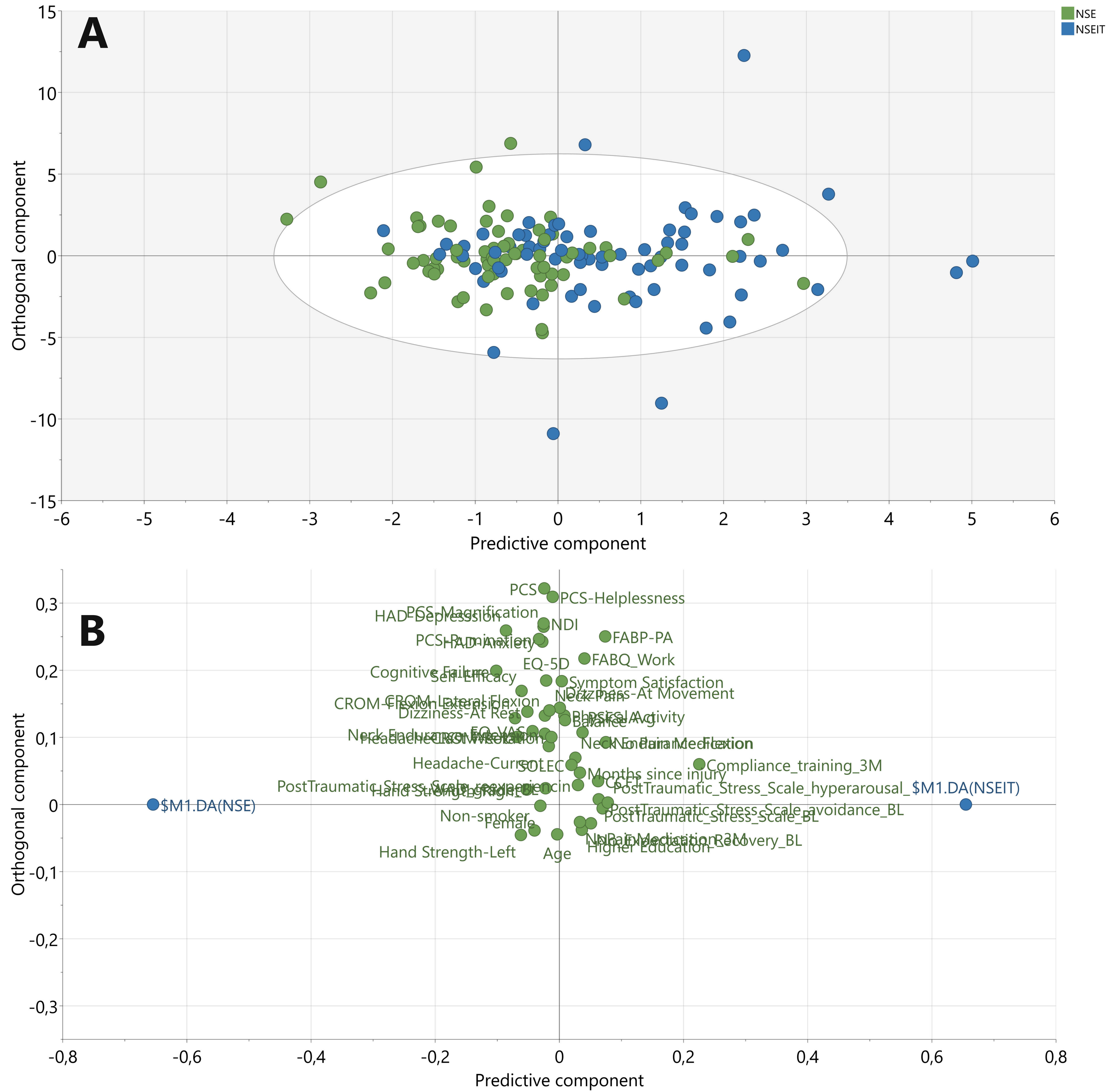

A
NSE
NSEIT
Orthogonal component
Predictive component
B
PCS
PCS-Helplessness
PCS-Magnification
HAD-Depression
NDI
PCS-Rumination
HAD-Anxiety
FABP-PA
FABQ_Work
EQ-5D
Cognitive Failure
Self-Efficacy
Symptom Satisfaction
Dizziness-At Movement
Neck Pain
CROM-Lateral Flexion
CROM-Flexion-extension
Dizziness-At Rest
Physical Activity
Balance
EQ-VAS
Headache-Current
SOLEC
Months since injury
Compliance_training_3M
PostTraumatic_Stress_Scale_hyperarousal_
$M1.DA(NSEIT)
$M1.DA(NSE)
PostTraumatic_Stress_Scale_avoidance_BL
PostTraumatic_Stress_Scale_BL
Non-smoker
Female
Hand Strength-Left
Age
Higher Education
