# Supplementary material for: Factors associated with 1-year health-related quality of life after neck-specific exercise in chronic whiplash-associated disorders: an exploratory secondary analysis of a randomized controlled trial
Source: BMC Musculoskelet Disord. 2026 Oct 13;27:871. doi: 10.1186/s12891-026-10581-x (PMC13670587; doi:10.1186/s12891-026-10581-x)
Supplement: Supplementary file 10 [file 12891_2026_10581_MOESM7_ESM.pdf]

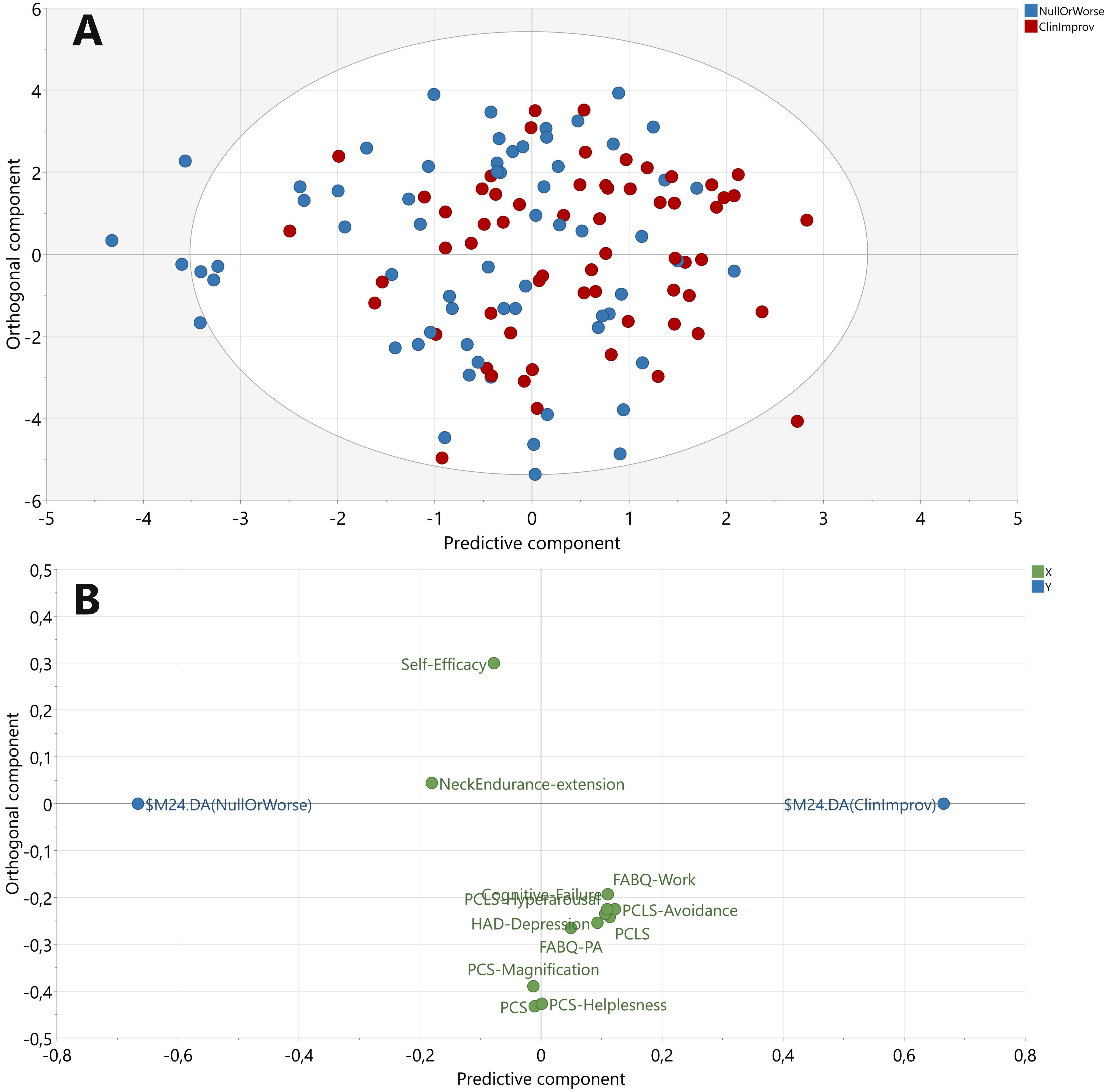
A
NullOrWorse
ClinImprov
Orthogonal component
Predictive component
B
X
Y
Self-Efficacy
NeckEndurance-extension
$M24.DA(NullOrWorse)
$M24.DA(ClinImprov)
FABQ-Work
Cognitive-Failure
PCLS-Hyperarousal
PCLS-Avoidance
HAD-Depression
PCLS
FABQ-PA
PCS-Magnification
PCS
PCS-Helplesness
Orthogonal component
Predictive component
