# Supplementary material for: Factors associated with 1-year health-related quality of life after neck-specific exercise in chronic whiplash-associated disorders: an exploratory secondary analysis of a randomized controlled trial
Source: BMC Musculoskelet Disord. 2026 Oct 13;27:871. doi: 10.1186/s12891-026-10581-x (PMC13670587; doi:10.1186/s12891-026-10581-x)
Supplement: Supplementary file 11 [file 12891_2026_10581_MOESM8_ESM.pdf]

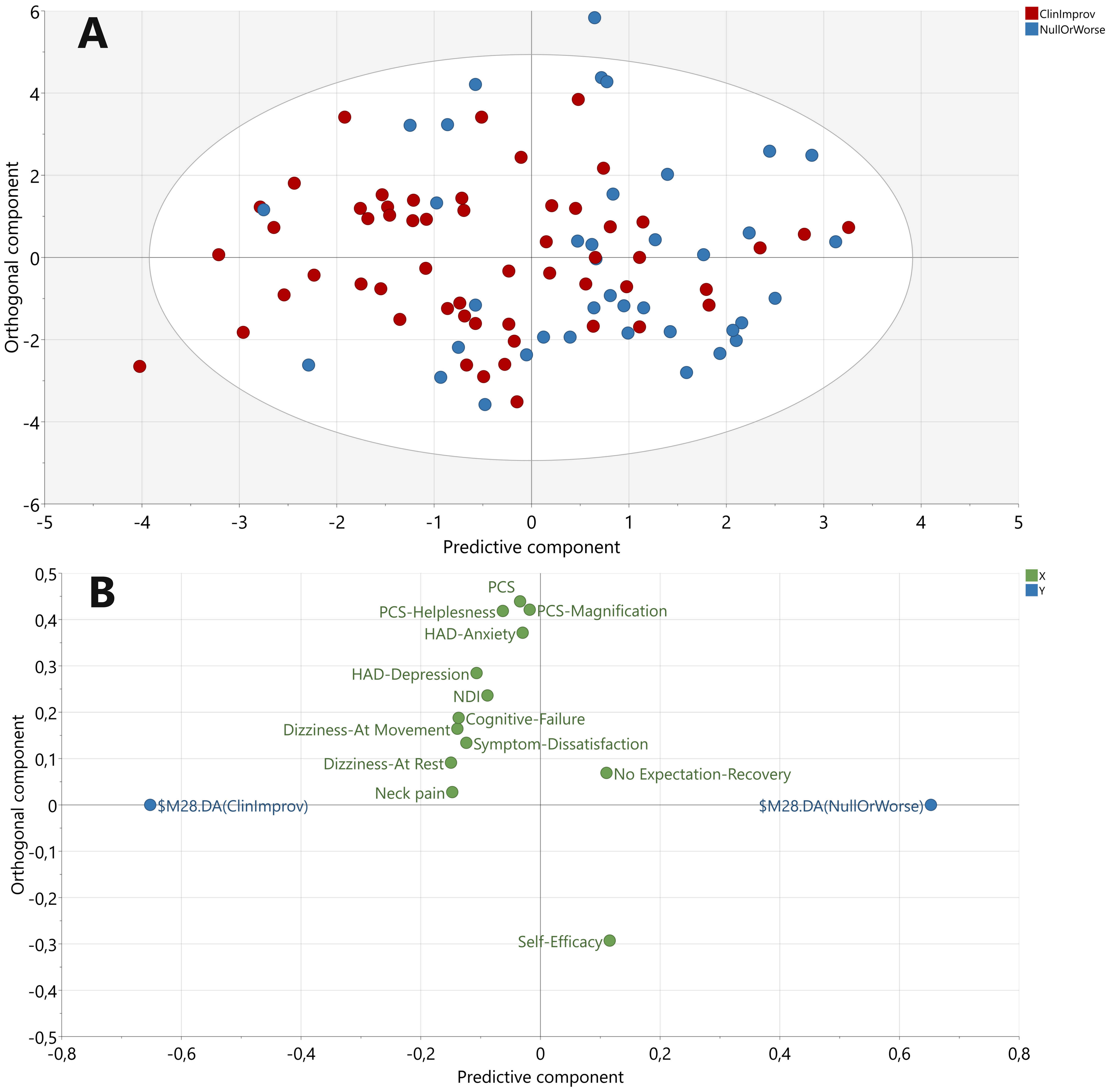

A
ClinImprov
NullOrWorse
Orthogonal component
Predictive component
B
X
Y
PCS
PCS-Helplesness
PCS-Magnification
HAD-Anxiety
HAD-Depression
NDI
Cognitive-Failure
Dizziness-At Movement
Symptom-Dissatisfaction
Dizziness-At Rest
No Expectation-Recovery
Neck pain
$M28.DA(ClinImprov)
$M28.DA(NullOrWorse)
Self-Efficacy
Orthogonal component
Predictive component
