# Supplementary material for: Factors associated with 1-year health-related quality of life after neck-specific exercise in chronic whiplash-associated disorders: an exploratory secondary analysis of a randomized controlled trial
Source: BMC Musculoskelet Disord. 2026 Oct 13;27:871. doi: 10.1186/s12891-026-10581-x (PMC13670587; doi:10.1186/s12891-026-10581-x)
Supplement: Supplementary file 12 [file 12891_2026_10581_MOESM9_ESM.pdf]

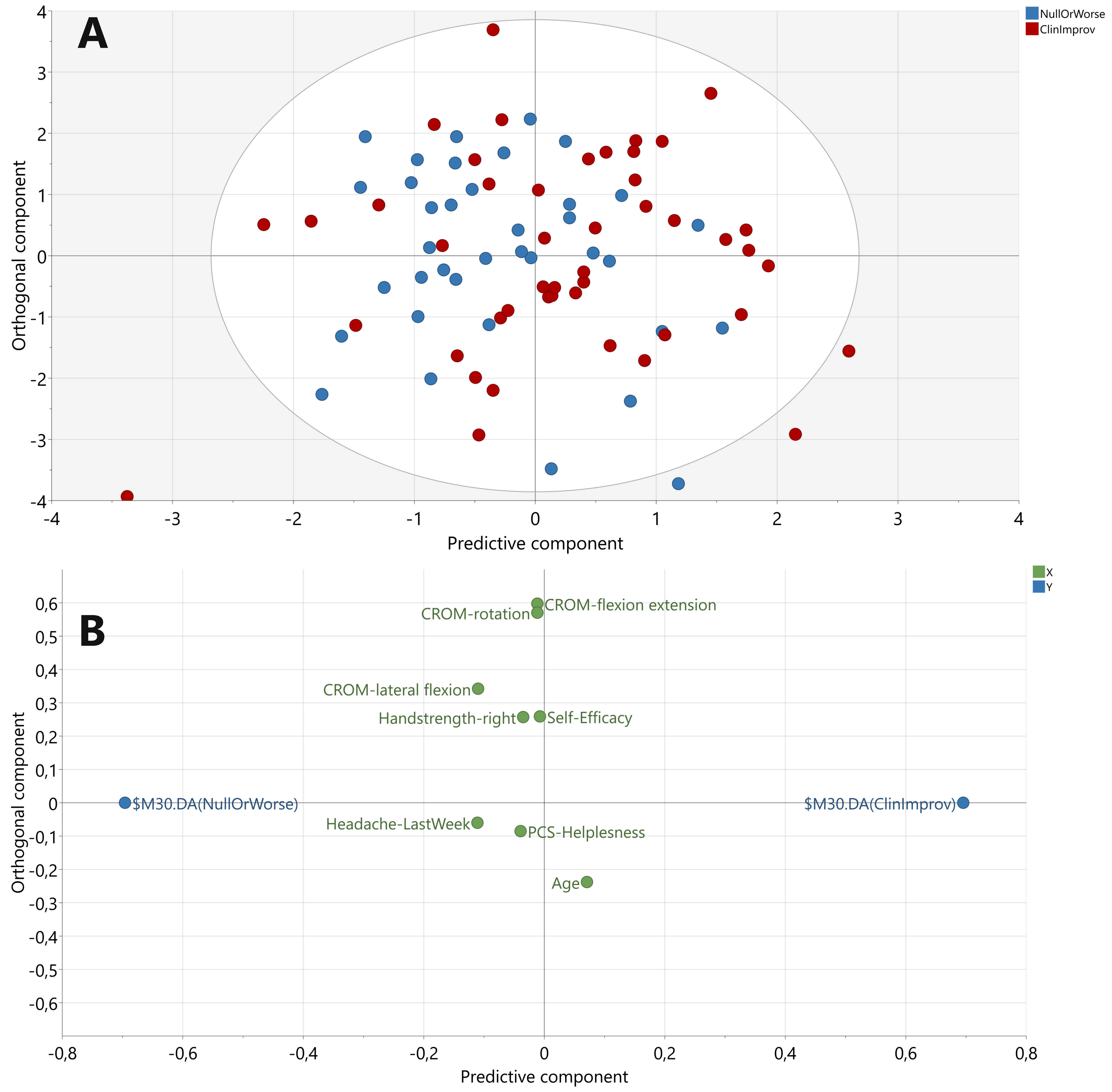

A
NullOrWorse
ClinImprov
Orthogonal component
Predictive component
B
X
Y
CROM-flexion extension
CROM-rotation
CROM-lateral flexion
Handstrength-right
Self-Efficacy
$M30.DA(NullOrWorse)
$M30.DA(ClinImprov)
Headache-LastWeek
PCS-Helplesness
Age
Orthogonal component
Predictive component
