# Supplementary material for: Factors associated with 1-year health-related quality of life after neck-specific exercise in chronic whiplash-associated disorders: an exploratory secondary analysis of a randomized controlled trial
Source: BMC Musculoskelet Disord. 2026 Oct 13;27:871. doi: 10.1186/s12891-026-10581-x (PMC13670587; doi:10.1186/s12891-026-10581-x)
Supplement: Supplementary file 13 [file 12891_2026_10581_MOESM13_ESM.docx]

**SUPPORTING (Supplementary; S) INFORMATION:**

S1. CONSORT 2010 checklist

S2. SPIRIT checklist

S3. Supplementary table. Missing data table for used variables.

S4. Comparison between the NSEIT and NSE group baseline to 3-month follow-up. Orthogonal partial least squares discriminant analysis (OPLS-DA) model of NSE and NSEIT including the two dependent variables (EQ-VAS and EQ-5D) and the change score (baseline to 3 months) of all independent variables. No clear separation of the NSEIT and NSE groups were seen. The x-axis depicts the predictive component most important for separating groups, while the y-axis represents the orthogonal component showing in-group variation, R^2^ = 0.21, Q^2^ = -0.12, CV-ANOVA p = 1.

S5. Comparison between the NSEIT and NSE group baseline to 15-month follow-up. Orthogonal partial least squares discriminant analysis (OPLS-DA) model of NSE and NSEIT including the two dependent variables (EQ-VAS and EQ-5D) and the change score (baseline to 15 months) of all independent variables. No clear separation of the NSEIT and NSE groups were seen. The x-axis depicts the predictive component most important for separating groups, while the y-axis represents the orthogonal component showing in-group variation, R^2^ = 0.22, Q^2^ = 0.01, CV-ANOVA p = 1.

S6. Comparison between the NSEIT and NSE group 3- to 15-month follow-up. Orthogonal partial least squares discriminant analysis (OPLS-DA) model of NSE and NSEIT including the two dependent variables (EQ-VAS and EQ-5D) and the change score (3 to 15 months) of all independent variables. No clear separation of the NSEIT and NSE groups were seen. The x-axis depicts the predictive component most important for separating groups, while the y-axis represents the orthogonal component showing in-group variation, R^2^ = 0.17, Q^2^ = -0.10, CV-ANOVA p = 1.

## S7. Comparison of patients with or without clinical improvement in EQ-5D at 3-month follow-up based on baseline variables. OPLS-DA model of clinically improved individuals (≥ 0.03 increment in EQ-5D, based on Soer et al. 2012) compared to individuals with non-effect/worse EQ-5D (≤ 0) at 3-month follow-up, based on the baseline variables. No clear separation of the improved vs. not improved individuals could be seen. The x-axis depicts the predictive component most important for separating groups, while the y-axis represents the orthogonal component showing in-group variation, R^2^ = 0.13, Q^2^ = 0.02, CV-ANOVA p > 0.05.

## S8. Comparison of patients with or without clinical improvement in EQ-VAS at 3-month follow-up based on baseline variables. OPLS-DA model of clinically improved individuals (≥ 10.5 increment in EQ-VAS, based on Soer et al. 2012) compared to individuals with non-effect/worse EQ-VAS (≤ 0) at 3-month follow-up, based on the baseline variables. No clear separation of the improved vs. not improved individuals could be seen. The x-axis depicts the predictive component most important for separating groups, while the y-axis represents the orthogonal component showing in-group variation, R^2^ = 0.14, Q^2^ = -0.05, CV-ANOVA p > 0.05.

## S9. Comparison of patients with or without clinical improvement in EQ-VAS at 15-month follow-up based on baseline variables. OPLS-DA model of clinically improved individuals (≥ 10.5 increment in EQ-VAS, based on Soer et al. 2012) compared to individuals with non-effect/worse EQ-VAS (≤ 0) at 15-month follow-up, based on the baseline variables. No clear separation of the improved vs. not improved individuals could be seen. The x-axis depicts the predictive component most important for separating groups, while the y-axis represents the orthogonal component showing in-group variation, R^2^ = 0.06, Q^2^ = -0.12, CV-ANOVA p > 0.05.

## S10. Permutation plot of the OPLS-DA model discriminating between participants with or without clinical relevant improvement in EQ-5D at the 15 months follow-up. A total of 100 permutations were performed. The original model is on the right while permuted models are on the left. The original model yielded higher R² and Q² values than the permuted models, and the Q² regression line had a negative intercept. This result suggests that the observed class separation was unlikely to be explained by chance alone.

## S11. Sensitivity analysis of findings with prediction of clinical improvement in EQ-5D at 15-month follow-up. An OPLS-DA model similar to Figure 5 was constructed in which the base-line value of EQ-5D-3L was included to test for its relation to clinical outcome. The figure shows the loading plot in which EQ-5D-3L baseline value is seen in the first quadrant and as such indicates that higher baseline EQ-5D-3L is negatively associated with clinical improvement (blue indicator to the right).

## S12. Supplementary table. Comparison of results with the study by Ludvigsson et al. 2019.
